# Single-cell profiling of the antigen-specific response to BNT162b2 SARS-CoV-2 RNA vaccine

**DOI:** 10.1038/s41467-022-31142-5

**Published:** 2022-06-16

**Authors:** Kevin J. Kramer, Erin M. Wilfong, Kelsey Voss, Sierra M. Barone, Andrea R. Shiakolas, Nagarajan Raju, Caroline E. Roe, Naveenchandra Suryadevara, Lauren M. Walker, Steven C. Wall, Ariana Paulo, Samuel Schaefer, Debolanle Dahunsi, Camille S. Westlake, James E. Crowe, Robert H. Carnahan, Jeffrey C. Rathmell, Rachel H. Bonami, Ivelin S. Georgiev, Jonathan M. Irish

**Affiliations:** 1 Department of Pathology, Microbiology, and Immunology, Vanderbilt University Medical Center, Nashville, TN 37232 USA; 2 Vanderbilt Vaccine Center, Nashville, TN 37232 USA; 3 Department of Medicine, Division of Rheumatology and Immunology, Vanderbilt University Medical Center, Nashville, TN 37232 USA; 4 Human Immunology Discovery Initiative of the Vanderbilt Center for Immunobiology, Nashville, TN 37232 USA; 5 Vanderbilt Institute for Infection, Immunology, and Inflammation, Nashville, TN 37232 USA; 6 Department of Cell and Developmental Biology, Vanderbilt University, Nashville, TN 37232 USA; 7 Vanderbilt-Ingram Cancer Center, Vanderbilt University Medical Center, Nashville, TN 37232 USA; 8 Department of Pediatrics, Vanderbilt University Medical Center, Nashville, TN 37232 USA; 9 Vanderbilt Program in Computational Microbiology and Immunology, Nashville, TN 37232 USA

**Keywords:** Viral infection, Translational research, RNA vaccines, SARS-CoV-2, Lymphocyte differentiation

## Abstract

RNA-based vaccines against SARS-CoV-2 have proven critical to limiting COVID-19 disease severity and spread. Cellular mechanisms driving antigen-specific responses to these vaccines, however, remain uncertain. Here we identify and characterize antigen-specific cells and antibody responses to the RNA vaccine BNT162b2 using multiple single-cell technologies for in depth analysis of longitudinal samples from a cohort of healthy participants. Mass cytometry and unbiased machine learning pinpoint an expanding, population of antigen-specific memory CD4^+^ and CD8^+^ T cells with characteristics of follicular or peripheral helper cells. B cell receptor sequencing suggest progression from IgM, with apparent cross-reactivity to endemic coronaviruses, to SARS-CoV-2-specific IgA and IgG memory B cells and plasmablasts. Responding lymphocyte populations correlate with eventual SARS-CoV-2 IgG, and a participant lacking these cell populations failed to sustain SARS-CoV-2-specific antibodies and experienced breakthrough infection. These integrated proteomic and genomic platforms identify an antigen-specific cellular basis of RNA vaccine-based immunity.

## Abstract (web summary)

Vaccination against COVID-19 has shown activation of different immune cell types. Here the authors characterise the immune response to the SARS-CoV-2 mRNA vaccine using longitudinal CyTOF single cell approaches to characterise antigen specific B and T-cell responses promoted by this vaccine.

## Introduction

In December 2019, a novel coronavirus strain designated severe acute respiratory distress syndrome coronavirus 2 (SARS-CoV-2) was identified in Wuhan, China. A global pandemic ensued that has resulted in over 275 million cases and 5 million deaths of coronavirus disease 2019 (COVID-19) to date^1^. B cells, T cells, and other leukocytes undergo significant shifts upon SARS-CoV-2 infection that may contribute to anti-viral immunity and protective antibodies^2–8^. The development of viral neutralizing antibodies following infection has been associated with an increased abundance of Th1-like CD8^+^ and CD4^+^ cells, circulating CD4^+^ T follicular helper cells (cTfh), and activated T cells^3,4^. While multiple therapies, such as dexamethasone^9,10^, baracitinib^11,12^, tocilizumab^12,13^, and neutralizing monoclonal antibodies^14,15^ have emerged as treatments for severe COVID-19 disease, preventive measures to develop coronavirus immunity on a population-scale are of upmost importance. To address this need, vaccines formulated with the pre-fusion stabilized SARS-CoV-2 Spike (S) protein were developed to induce protection from COVID-19 infection or development of severe disease^16–20^. Globally, nearly 9 billion doses of various COVID-19 vaccines have been administered^1^.

Messenger ribonucleic acid (mRNA)-based vaccines represent a promising class of vaccines that offer protection from COVID-19 as well as potentially a wide range of emerging infectious diseases^21,22^. These vaccines introduce minimal genetic information to express viral antigens of interest^21^ while mimicking some features of infection with RNA viruses, such as SARS-CoV-2^23^. Several groups have explored the immunologic response to SARS-CoV-2 mRNA vaccines using both systems^24–26^ and T-cell centric approaches^27–30^ and elevated myeloid and T-cell responses have been identified following vaccination and which corresponded to serologic antibody responses. These studies, however, have not provided sufficient single-cell depth of analysis to directly identify and characterize antigen-specific cells that respond to vaccination and mechanisms of mRNA-based formulations. It is also not well established how pre-existing immunity to endemic coronaviruses impacts the B-cell and antibody response to SARS-CoV-2 vaccination and how the antibody repertoire may evolve over time

A major challenge to studies of immune responses to emerging diseases is the reliable identification of antigen-specific cells. While MHC tetramers and other tracking agents can identify pre-determined subsets of antigen-specific cells, a systemic and unbiased view of SARS-CoV-2 antigen-responsive cells is needed. Single-cell machine learning analysis tools including the Tracking Responders Expanding (T-REX) algorithm^31^ and Linking B-cell Receptor to Antigen specificity through sequencing (LIBRA-seq)^32^ combined with whole transcriptome RNA-seq can address this need to provide unbiased identification and characterization of immune cells reacting to infection or vaccination. These single-cell approaches identify rare cells that specifically expand following vaccination or infection that can be overlooked when analyzing cellular populations in bulk. Proteomic signatures identified by T-REX can be combined with Marker Enrichment Modeling (MEM)^33^ to develop strategies to physically isolate the responding cell subset using fluorescence-activated cell sorting (FACS) enabling in vitro validation. LIBRA-seq identifies antigen-binding B cells and their associated B-cell receptor (BCR) sequences in parallel with single-cell RNA-seq to identify and characterize B-cell subsets based on transcriptional profiles^32^. Adaptation and evolution of antibody isotypes and antigen-binding properties provide further insight into the development of B-cell responses and antigen specificity.

In this work, we employ unbiased approaches to identify and track the development of antigen-specific T and B cells in longitudinal samples from healthy participant recipients of the BNT162b2 RNA-based SARS-CoV-2 vaccine, including a participant with a subsequent breakthrough SARS-CoV-2 infection. We also identify expanding and metabolically active S protein-specific, CD4^+^ and CD8^+^ T-cell populations following vaccination and confirm antigen specificity in vitro assays. In parallel, single-cell LIBRA-seq and RNA-seq of coronavirus S protein-binding B cells establish the evolution of cross-reactive to antigen-specific B cells with public coronavirus antibody sequences over time. Antigen-specific T and B cells correlate and associate with a long-lasting IgG response and are not seen in a participant with subsequent breakthrough infection. These cell and antibody associations may drive further efforts to predict vaccine effectiveness and identify mechanisms of protection.

## Results

### Mass cytometry identifies vaccine-induced CD4^+^ and CD8^+^ ICOS^+^CD38^+^CXCR5^−^ subsets

**Fig. 1:**
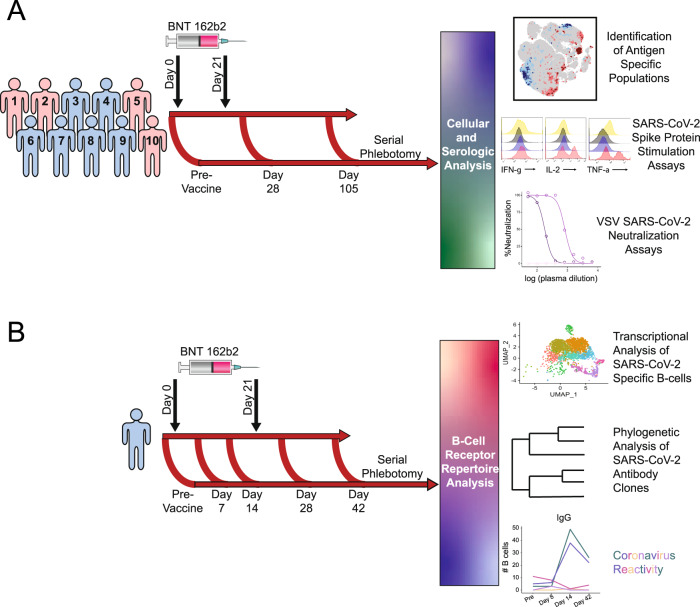
Schematic of vaccination schedule and sample collection. All participants were vaccinated with BNT162b2 on days 0 and 21 and samples were collected early in 2021. **A** 10 healthy participants underwent serial phlebotomy that was performed pre-vaccination (day −3 to 0), on day 28–30, and on day 105-108. PBMCs were isolated at each time point, and citrated plasma was stored when possible. PBMCs from these participants were utilized for CyTOF and in vitro stimulation studies. Plasma was used both for SARS-CoV-2 ELISAs and vesicular stomatitis virus pseudoneutralization assays. **B** A single healthy participant underwent serial phlebotomy pre-vaccination and on days 8, 14, 28, and 42. PBMCs and citrated plasma were isolated at each time point and used for transcriptional analysis of SARS-CoV-2-specific B cells.

**Fig. 2:**
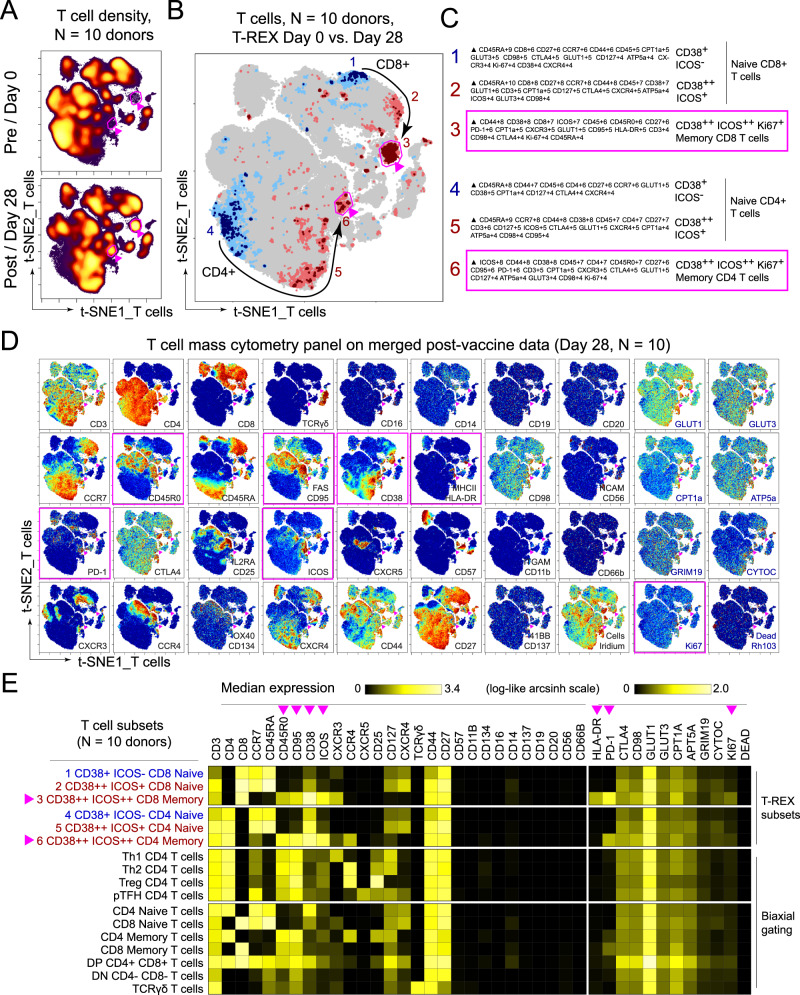
Immune phenotyping of BNT162b2 responding T cells. PBMCs collected from study participants (*n* = 10) pre-vaccination (day 0), and day 28 post-vaccination (7 days post-boost) were analyzed individually by mass cytometry in one setting to avoid batch effects, and data were concatenated for analysis. **A** CD3^+^ T cells from 10 participants were pooled into two sets, one for pre-vaccination (taken at day 0) and one for post-vaccination (day 28). Cell density for each set is shown on the t-SNE axes. **B** T-REX analysis of the CD3^+^ T cells from the 10 participants is shown. A central t-SNE, performed only using cell surface markers, shaded by T-REX change depicts phenotypically similar cells in regions of great expansion (dark red, ≥95% from post-vaccination day 28) or great contraction (dark blue, ≥95% from pre-) over time following SARS-CoV-2 vaccination. Red or blue population interpretations are shown for major expanding or contracting populations identified by T-REX. **C** MEM labels show enriched protein features for several populations of great expansion or contraction. All measured features were included in MEM enrichment labels, which only show features enriched by at least +4 on a scale from 0 to 10. Pink boxes are around the MEM labels for the CD4^+^ and CD8^+^ memory T-cell clusters that are greatly enriched for ICOS and CD38 protein and expanded greatly following vaccination. **D** Each protein marker is shown on t-SNE axes, with proteins that were enriched on CD4^+^ICOS^+^CD38^+^ and CD8^+^ICOS^+^CD38^+^ cells in pink boxes. A rainbow intensity scale indicates expression levels with red representing high and dark blue representing low expression. Protein names in blue indicate functional features that were not used in t-SNE analysis, including metabolic markers, Rhodium, and Ki67. Surface proteins in black were used in t-SNE analysis. **E** Heat maps show all markers measured by mass cytometry for cell populations as determined by T-REX or expert gating. Cell labels in red were defined by T-REX as expanding and blue were defined as contracting by T-REX. Black cell labels were expert gated. Protein markers enriched in CD4^+^ICOS^+^CD38^+^ and CD8^+^ICOS^+^CD38^+^ cells are indicated with pink arrows.

The effect of BNT162b2 immunization was explored on recipient T-cell populations in a cohort of ten healthy participants with samples collected at Vanderbilt University Medical Center in late 2020 and early 2021 following early Alpha and Beta variants but prior to the onset of Delta and later variants (Fig. 1). Each participant reported to have not been previously infected with SARS-CoV-2 and none had detectable neutralizing antibody titer prior to vaccination. Participants had an average age of 41.8 ± 6.3 years (range 35–57). Six participants were male, and nine participants identified as having Caucasian ancestry. One participant was lost to follow-up between days 105–180. By collecting longitudinal peripheral blood samples before immunization, one week following a second dose immunization (day 28), and at an additional time point approximately three months later (day 105), we captured signatures of the initial immune response to BNT162b2 as well as lasting immunity (Fig. 1A). T-cell populations in the pre-vaccination and post-boost samples were initially examined by mass cytometry using a Helios cytometry by time-of-flight mass spectrometry (CyTOF) instrument and a panel of antibodies focused on T-cell immune and metabolic phenotypes (Supplementary Table 1). Cell density plots of concatenated data of T cells from the pre-vaccination and post-boost samples were visualized using t-SNE dimensionality reduction (Fig. 2A). Because MHC-peptide tetramer staining reagents were not available to directly identify expanding antigen-specific S protein-reactive CD4^+^ and CD8^+^ T cells, data were analyzed using the recently developed T-REX machine learning algorithm^31^. By comparing changes in small k-nearest neighbor cell groupings, T-REX specifically identifies populations of cells with the greatest degrees of expansion or contraction from pre- to post-vaccination. In the case of viral infections, these expanding populations were preferentially enriched for virus-specific cells^31^. This approach sets aside most peripheral blood T cells, which were unchanged, to instead focus on those populations of T cells in phenotypically distinct regions whose abundance was among the ≥95% most increased or decreased in the initial 7 days following vaccination.

T-REX identified related populations of CD4^+^ and CD8^+^ T cells that expanded by ≥95% following vaccination and one population each that contracted by ≥95% (Fig. 2B). Across the cohort, changes in abundance of these cell populations were widely, but not universally, shared in T-REX of individual participant samples (Supplementary Data 1, Supplementary Fig. 1). MEM and specific antibody staining patterns established the protein marker expression patterns characteristic of each population (Fig. 2C, D). The most expanded populations were CCR7^-^ CD45R0^+^ CD4^+^ and CD8^+^ T-cell populations that were negative for CXCR5, positive for PD-1, and highly co-expressed Inducible T-cell costimulatory (ICOS) and CD38 (Fig. 2E). Consistent with extensive expansion, these cells were the most proliferative cell subset based on Ki67 positivity and were highly metabolically active based on the co-expression of transporters for glucose (GLUT1), amino acids (CD98), and lipids (CPT1a). These cells, thus, reflected an activated memory T-cell phenotype. The T-cell populations with decreasing abundance, in contrast, were CD45RA^+^ ICOS^−^ and phenotypically characterized as naive.

### ICOS^+^CD38^+^ T-cell subsets respond specifically to SARS-CoV-2 S protein antigen

**Fig. 3:**
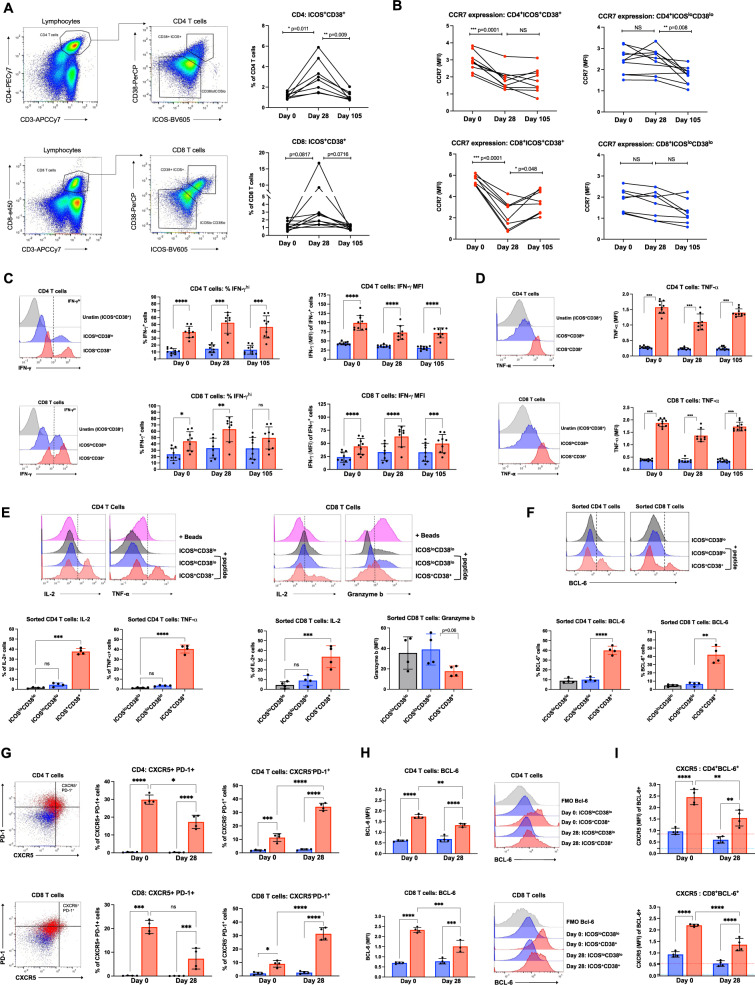
Functional analysis of ICOS^+^CD38^+^ CD4^+^ and CD8^+^ T cells and response to SARS-CoV-2 Spike antigen. PBMCs collected from study participants (*n* = 10) pre-vaccination (day 0), day 28 post-vaccination (7 days post-boost), and day 105 post-vaccination were examined for ICOS, CD38, and other markers by fluorescence flow cytometry individually in one setting to avoid batch variation. **A** CD4^+^ T cells were gated from lymphocytes and defined as ICOS^+^CD38^+^ or ICOS^lo^CD38^lo^. Percentages of CD4^+^ICOS^+^CD38^+^ T cells were quantified and data from each participant is connected by lines. **B** Mean fluorescence intensity (MFI) of CCR7 within CD4^+^ICOS^+^CD38^+^, CD8^+^ICOS^+^CD38^+^, CD4^+^ICOS^lo^CD38^lo^, or CD8^+^ICOS^lo^CD38^lo^ T cells. **C**, **D** PBMCs were stimulated with PMA/ionomycin and examined for cytokine production. T cells defined as ICOS^+^CD38^+^ (red) or ICOS^lo^CD38^lo^ (blue) were examined for **C** IFN-γ or **D** TNF. Representative samples on the left and individual participants are shown on right. **E** Day 28 PBMC samples were stimulated with activation beads or T cells were sorted based on CD38 and ICOS expression. Sorted T cells were labeled with CellTrace Violet (CTV) to track and stimulated with T-cell (CD3)-depleted autologous PBMCs ± SARS-CoV-2 Spike peptide pool (+ peptide, blue and red). Sorted T-cell populations were analyzed after 48 h for IL-2, TNF, and Granzyme b in **F**. BCL6 expression in sorted T-cell populations, stimulated as in **E**. **G** Tfh markers CXCR5 and PD-1 compared on day 0 and 28 samples. **H** Unstimulated days 0 and 28 ICOS^+^CD38^+^ and ICOS^lo^CD38^lo^ T cells were analyzed for BCL6 expression (FMO; fluorescence-minus-one control for BCL6). **I** CXCR5 MFI within BCL6^+^ T cells. FMO control for BCL6 in ICOS^lo^CD38^lo^ T cells is shown by the blue dotted line, and by the red dotted line for ICOS^+^CD38^+^ T cells. **B** Significance was determined by paired two-way ANOVA mixed-effects model. **E**, **F** Repeated measures one-way ANOVA. **C**, **D**, **G**–**I** Two-way ANOVA with Sidak’s multiple comparisons test. Representative samples are shown and each data point represents an individual participant. All error bars ± SD. ns = not significant, *p < 0.5, **p < 0.005, ***p < 0.0005, ****p < 0.00005.

The selective enrichment following vaccination of CD4^+^ICOS^+^CD38^+^ and CD8^+^CXCR5^−^PD1^+^ T cells suggested they may have specifically recognized and had expanded in response to vaccine encoded viral S protein. To test SARS-CoV-2 specificity and further characterize the ICOS^+^CD38^+^ T-cell populations, fluorescence flow cytometry was used to characterize cell subsets. Similar to the findings by mass cytometry, CD4^+^ICOS^+^CD38^+^, and CD8^+^ ICOS^+^CD38^+^ cells were present in pre-immunized samples as ~1–2% of each T-cell subset (Fig. 3A). Importantly, these cell populations increased in the post-second dose sample and returned to initial frequencies at a later sample collection point. The ICOS^+^CD38^+^ post-boost populations also downregulated CCR7 expression (Fig. 3B) consistent with mass cytometry findings and migration from lymph nodes, while this did not occur in ICOS^−^CD38^−^ T cells from the same samples. To investigate the functional capabilities of ICOS^+^CD38^+^ T cells, samples were stimulated with PMA, and ionomycin and cytokines were measured. At all collected timepoints, a significantly greater frequency of ICOS^+^CD38^+^ than ICOS^−^CD38^−^ T cells produced IFN-γ and TNF, and per cell levels of cytokine were greater in ICOS^+^CD38^+^ T cells based on intracellular cytokine mean fluorescence intensity (Fig. 3C, D). These data demonstrate a greater inherent cytokine-producing capability of ICOS^+^CD38^+^ than ICOS^−^CD38^−^ T cells.

To evaluate the antigen-specific response to SARS-CoV-2 S antigen, PBMCs from each time point were incubated with recombinant SARS-CoV-2 S1 domain of the S protein to mimic exposure to SARS-CoV-2 virus. T-cell activation beads were compared as a positive T-cell activating control and cytokine responses were measured in the culture supernatant. Interestingly, samples from immunized participants showed a wide distribution for IFN-γ and TNF production following stimulation, with some clear positive samples and others similar to the unstimulated controls (Supplementary Fig. 2A, B). IL-2, IL-4, and IL-17a were unaffected by the stimulation of T cells with S1 protein (Supplementary Fig. 2C–E). A subset of samples from all three time points produced IL-6 in response to S1 protein, suggesting cross-reactivity with prior coronavirus exposures (Supplementary Fig. 2F). Especially notable were the late collection time points in which three participant samples produced more IL-6 when incubated with S1 protein than samples stimulated with activation beads. Samples from day 105 also showed an increase in the frequency of IFN-γ-producing CD8^+^ T cells when restimulated with S1 protein after 11 days of culture (Supplementary Fig. 2G).

To directly test antigen specificity and further characterize the T cells identified by T-REX, CD4^+^ICOS^+^CD38^+^, CD8^+^ICOS^+^CD38^+^, CD4^+^ICOS^−^CD38^−^, and CD8^+^ICOS^−^CD38^−^ cells were isolated by cell sorting from four participant samples. Purified cells were then labeled with CellTrace Violet (CTV) to allow sorted cells to be tracked following when mixed with CD3-depleted autologous PBMCs and left unstimulated or stimulated with SARS-CoV-2 S protein-peptide pool (Supplementary Fig. 2H). After 48 h stimulation, ~40% of CTV^+^ ICOS^+^CD38^+^ CD4^+^ T cells produced IL-2 and TNF in response to SARS-CoV-2 S peptide stimulation, while only ~5% of peptide-stimulated ICOS^−^CD38^−^ T cells from the same sample produced cytokines when stimulated with SARS-CoV-2 S peptides (Fig. 3E). CTV^+^ CD8^+^ ICOS^+^CD38^+^ T cells also produced significant IL-2 in response to peptide stimulation but had decreased Granzyme B, suggesting potential post-activation degranulation, and reduced cytotoxicity, or a shift in CD8^+^ T-cell populations to favor a less cytotoxic subset or phenotype.

Both CD4^+^ICOS^+^CD38^+^ and CD8^+^ICOS^+^CD38^+^ populations showed evidence of metabolic reprogramming and mTORC1 pathway activity appeared to increase based on elevated levels of phosphor-S6 (Supplementary Fig. 2I). Expression of the glucose transporter GLUT1, however, was unchanged or modestly reduced (Supplementary Fig. 2J). Because iron is required to support mitochondrial respiration and for effector and memory T-cell responses to vaccination^34^, the Transferrin receptor (CD71) was measured as an indicator of iron metabolism. CD71 expression was significantly higher on ICOS^+^CD38^+^ populations at all time points (Supplementary Fig. 2K). Together with CyTOF analyses that show elevated markers of an amino acid (CD98) and mitochondrial lipid (Cpt1a) uptake (Fig. 2D, E), these data suggest CD4^+^ICOS^+^CD38^+^ and CD8^+^ICOS^+^CD38^+^ T cells are metabolically active with evidence of high rates of mitochondrial oxidative phosphorylation characteristic of activated and memory T cells.

### ICOS^+^CD38^+^ T cells downregulate T follicular helper cell markers post-boost

T-REX-identified CD4^+^ and CD8^+^ T-cell populations shared some characteristics with cTfh such as ICOS and PD-1 expression. Interestingly, a portion of the sorted cells expressed the T follicular helper (Tfh) characteristic transcription factor BCL6 (Fig. 3F). However, Tfh also expresses the chemokine receptor CXCR5, whereas T-REX-identified cells lacked CXCR5 (Fig. 2D, E). Because cells were not sorted based on CXCR5 expression, we next determined whether the sorted cells were cTfh cells or had expanded from a cTfh-like cell type, we compared BCL6 and other Tfh cell markers on day 0 and day 28 (post-boost) samples. On day 0, ICOS^+^CD38^+^ T cells were significantly enriched for CXCR5 and PD-1 expression suggesting a mixed population of cTfh cells and other non-Tfh ICOS^+^CD38^+^ T cells (Fig. 3G). However, the cTfh-like population decreased by day 28. By contrast, ICOS^+^CD38^+^ T cells that were negative for CXCR5 but expressed PD-1, increased in frequency from day 0 to day 28 samples. Therefore, the T-REX identified T-cell subset likely expanded after antigenic stimulation more than traditional cTfh.

Interestingly, a greater frequency of both CD4^+^ICOS^+^CD38^+^ and CD8^+^ICOS^+^CD38^+^ T cells expressed B-cell lymphoma 6 protein (BCL6) than ICOS^−^CD38^−^ T cells at both day 0 and day 28 (Fig. 3H). There was, however, a significant decrease in BCL6 expression in the ICOS^+^CD38^+^ populations over time. The BCL6^+^ cell population was analyzed specifically, and BCL6^+^ICOS^+^CD38^+^ T cells had lower levels of CXCR5 expression on day 28 than on day 0, suggesting that either these cells downregulated CXCR5 or a CXCR5^−^ the population had selectively expanded (Fig. 3I). Furthermore, BCL6 expression seen in sorted peptide-stimulated cultures (Fig. 3F) could have originated from either CXCR5^−^ or CXCR5^+^ T cells. To address this, the proportions of BCL6 expression were compared in ICOS^+^CD38^+^ T cells that were either CXCR5^+^ or ^–^ (Supplementary Fig. 2L). As expected, BCL6 expression was enriched in CXCR5^+^ cells at all time points consistent with a cTfh phenotype. However, there was a slight decrease in BCL6 in CXCR5^+^ cells from day 0 to day 28 suggesting that traditional cTfh cells could be outcompeted by T-REX identified CXCR5^−^ T cells in response to antigen. The sorted population may thus reflect a mixture of T-cell subsets with expanding ICOS^+^CD38^+^ cells representative of a transitional Tfh, peripheral helper (Tph) memory^35^, or mixed population.

### BNT162b2 induces expansion of CD38^+^CD43^+^ plasmablasts

**Fig. 4:**
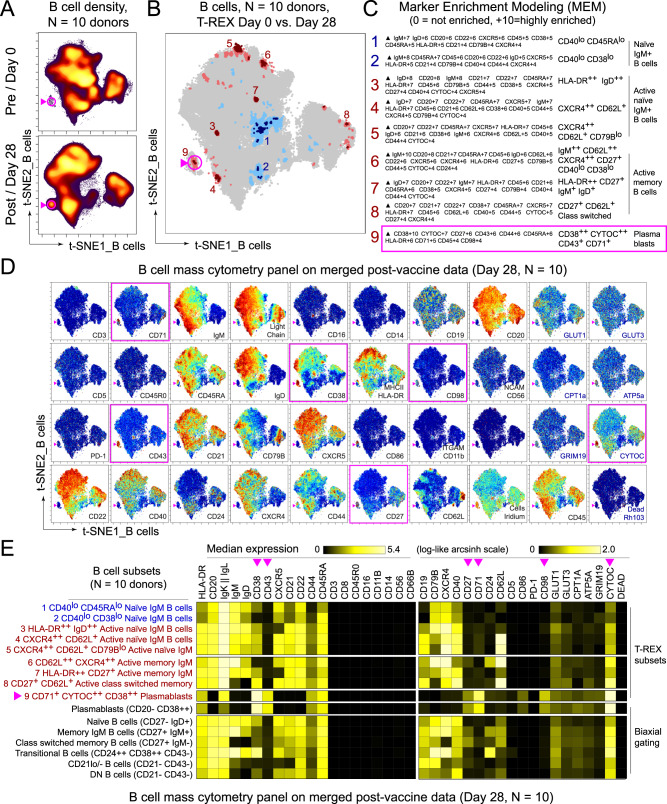
Immune phenotyping of BNT162b2 responding B cells. PBMCs collected from study participants (*n* = 10) pre-vaccination (day 0), day 28 post-vaccination (7 days post-boost) were individually by mass cytometry in one setting to avoid batch effects, and data were concatenated for analysis of expanded B-cell subsets and plasmablasts. **A** B cells from 10 participants were pooled into two sets, one for pre-vaccination (taken at day 0) and one for post-vaccination (day 28). Cell density for each set is shown on the t-SNE axes. **B** T-REX analysis of the B cells from the 10 participants is shown. A central t-SNE, performed only using cell surface markers, shaded by T-REX change depicts phenotypically similar cells in regions of great expansion (dark red, ≥95% from post-vaccination day 28) or great contraction (dark blue, ≥95% from pre-) over time following SARS-CoV-2 vaccination. Red or blue population interpretations are shown for major expanding or contracting populations identified by T-REX. **C** MEM labels show enriched protein features for several populations of great expansion or contraction. All measured features were included in MEM enrichment labels, which only show features enriched by at least +4 on a scale from 0 to 10. A pink box is around the MEM label for the plasmablast cluster that expanded greatly following vaccination. **D** Each protein marker is shown on t-SNE axes, with proteins that were enriched on the plasmablast population in pink boxes. A rainbow intensity scale indicates expression levels with red representing high and dark blue representing low expression. Protein names in blue indicate functional features that were not used in t-SNE analysis, including metabolic markers, Rhodium, and Ki67. Surface proteins in black were used in t-SNE analysis. **E** Heat maps show all markers measured by mass cytometry for cell populations as determined by T-REX or expert gating. Cell labels in red were defined by T-REX as expanding and blue were defined as contracting by T-REX. Black cell labels were expert gated. Protein markers enriched in plasmablasts are indicated with pink arrows.

The B-cell response to BNT162b2 was next examined using a similar approach as applied to the T-cell response. Peripheral blood samples from the same participant cohort were analyzed by mass cytometry using a panel of antibodies focused on B-cell populations and metabolic markers. T-REX analysis of equally sampled B cells from all individuals showed that while most B-cell populations did not change the following boost, there were nine phenotypically distinct areas of increased or decreased cell abundance (Fig. 4, Supplementary Data 1). Of these, seven populations were expanded and two contracted. The populations that expanded ≥95% from day 0 to day 28 included activated IgM^+^ B cells, memory B cells, and plasmablasts, while naive B cells greatly contracted over this time. While individual participants showed heterogeneity, these populations remained broadly evident (Supplementary Fig. 4). Using MEM to characterize cells identified by T-REX, the IgM^−^IgD^−^ plasmablast population was further defined as CD20^−^CD38^+^CD43^+^ and metabolically active, with high expression of the iron transporter CD71, CD98, and Cytochrome C (CYTOC) (Fig. 4C–E).

### Serologic response to BNT162b2 does not correlate to pre-existing immunity for endemic coronaviruses

**Fig. 5:**
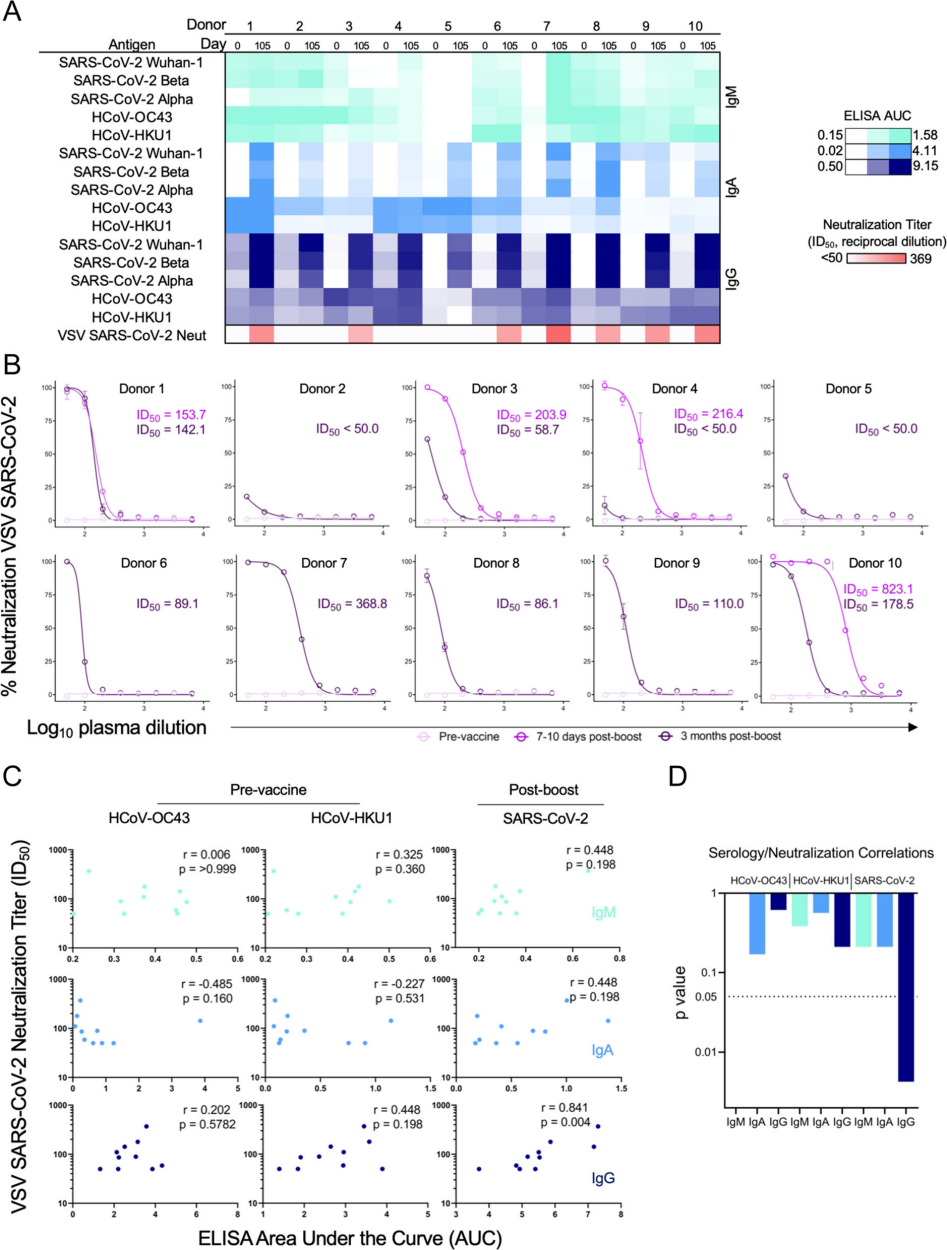
Serologic response induced by BNT162b2. Sera were analyzed from healthy participants (*n* = 10) pre-vaccination and at day 105 in one setting to avoid batch variation. **A** Log ELISA area under the curve (AUC) values from vaccine recipient plasma are depicted as a heatmap against spike proteins from SARS-CoV-2 Wuhan-1, SARS-CoV-2 Alpha, SARS-CoV-2 Beta, HCoV-OC43, and HCoV-HKU1 coronaviruses. The minimum signal is represented in white and the maximum signal is depicted by teal, blue, and navy blue for IgM, IgA, and IgG, respectively. At the bottom of the serology heatmap, the inhibitory dose at 50% neutralization (ID_50_) neutralization titer at the day 105 time point is depicted from zero neutralization (white) to the maximum Log ID_50_ value quantified in the participants. ELISAs were performed in technical duplicate and repeated once; neutralization assays were performed once in technical triplicate. **B** VSV SARS-CoV-2 neutralization curves of vaccine recipient plasma. Pre-vaccine, 7–10 days post-boost (collected only from participants 1, 3, 4, and 10), and 105 days post-boost are depicted in light pink, pink, and purple, respectively. %Neutralization (y-axis) is plotted as a function of log plasma dilution (*x* axis). Assays were done in technical triplicate. **C** Spearman correlations for serological responses as log ELISA AUC (x-axis) of patient cohort against pre-vaccination HCoV-OC43 (left), HCoV-HKU1 (middle), and post-boost SARS-CoV-2 (right) as a function of neutralization titer expressed as ID50 (*y* axis). IgM is depicted in teal (top), IgA is shown in blue (middle), and IgG is shown in navy blue (bottom). The respective rho and p values are shown in the top right of each plot. **D** Individual statistical significance comparison values are depicted as a bar graph for HCoV-OC43, HCoV-HKU1, and SARS-CoV-2 (left to right). IgM is depicted in teal, IgA is shown in blue, and IgG is shown in navy blue. All error bars are mean ± standard deviation.

BNT162b2-induced serological responses were measured by testing participant plasma samples from day 0, day 28, and day 105 for reactivity to S proteins from SARS-CoV-2 Wuhan-1, SARS-CoV-2 Beta, SARS-CoV-2 Alpha, HcoV-OC43, and HcoV-HKU1. Robust IgG responses were measured against SARS-CoV-2 Wuhan-1 by ELISA, with more varied IgM responses (Fig. 5A, Supplementary Fig. 5A). These patterns were generally consistent across the S proteins from circulating variants of concern Beta and Alpha. Interestingly, an IgA serological response was also evident in most participants. Most participants also displayed signatures of pre-existing immunity to the coronaviruses HcoV-OC43 and HcoV-HKU1 at day 0, consistent with their endemic nature among the human population^36^. Antibody levels to these endemic strains, however, did not change following BNT162b2 vaccination. To assess the functional serological response of each participant a replication-competent vesicular stomatitis virus (VSV) SARS-CoV-2^37^ Real-Time Cell Analysis neutralization assay^38^ was performed at both pre- and at multiple timepoints post-vaccination. While pre-vaccine samples exhibited no SARS-CoV-2 neutralization activity, most participants showed neutralizing activity on day 105, although 3 of the 10 participants showed only weak neutralization at that timepoint (Fig. 5B). Interestingly, a sharp decline in neutralization potency was observed for samples 3 months post-boost compared to day 7–8 post-boost in three of the four participants with multiple timepoints post-vaccination. Despite a modest trend for HcoV-HKU1 IgG, pre-existing antibody titers against HcoV-OC43 and HcoV-HKU1 did not correlate with SARS-CoV-2 neutralization potency 3 months post-vaccination (Fig. 5C). Among the serological variables that were tested, only SARS-CoV-2 IgG level 3 months post-boost had a significant correlation with VSV SARS-CoV-2 neutralization potency (Fig. 5C, D). Therefore, pre-existing endemic coronavirus antibodies did not appear to be a major determinant in the development of the antibody response against SARS-CoV-2 by BNT162b2 vaccination.

### Antigen selectivity of the SARS-CoV-2-reactive B-cell response to BNT162b2 increases over time

**Fig. 6:**
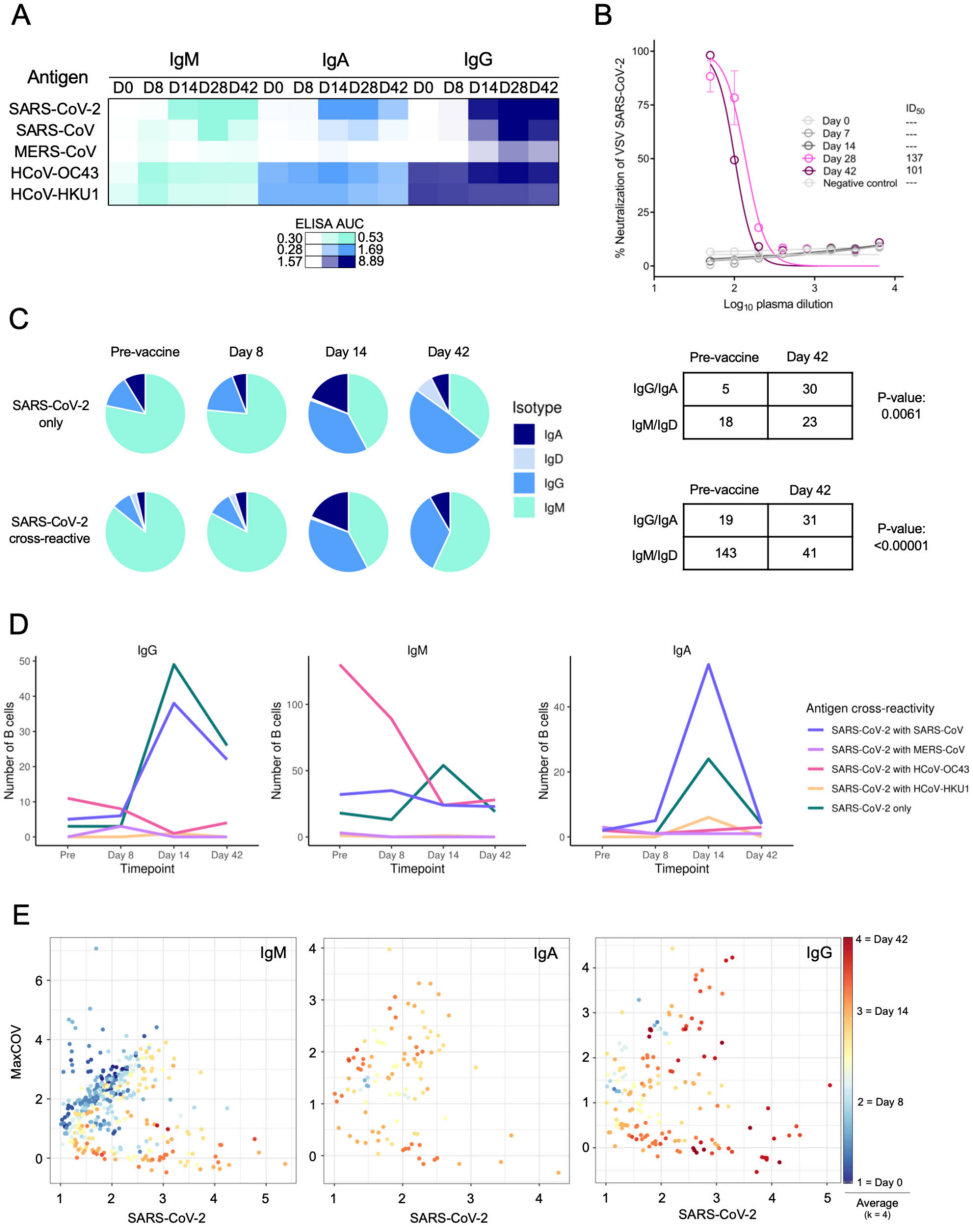
LIBRA-seq characterization of the antigen specificity of the SARS-CoV-2-reactive B-cell response to BNT162b2. A single participant with multiple longitudinal samples was analyzed for serological response and individual B-cell selectivity by LIBRA-seq. **A** Plasma log ELISA area under the curve (AUC) values are depicted as a heatmap against spike proteins for SARS-CoV-2, SARS-CoV, MERS-CoV, HCoV-OC43, and HCoV-HKU1. The minimum signal is represented in white and the maximum signal is depicted by teal, blue, and navy blue for IgM, IgA, and IgG, respectively. Assays were performed in technical duplicates and repeated once. **B** VSV SARS-CoV-2 neutralization of longitudinal timepoints. % Neutralization (y-axis) is plotted as a function of plasma dilution (*x* axis). The negative control sample, Day 0, Day 8, and Day 14 timepoints are depicted in gray, with Day 28 and Day 42 curves shown in pink and purple respectively. The inhibitory dose at 50% neutralization (ID_50_) values of each timepoint is denoted to the right of graph. Assays were performed in technical triplicate and performed once. **C** Pie charts representing: (top) SARS-CoV-2-specific B cells with an associated LIBRA-seq score ≥1 for SARS-CoV-2; and (bottom) SARS-CoV-2 cross-reactive B cells (bottom) with an associated LIBRA-seq score ≥1 for SARS-CoV-2 and for at least one other coronavirus antigen (MERS-CoV, SARS-CoV, HCoV-OC43, or HCoV-HKU1). For the pre-vaccine, day 8, day 14, and day 42 time points, the segments in each pie chart represent the number of antibody sequences with the isotypes IgD (light blue), IgM (teal), IgG (blue), IgA (navy blue). Also shown are a statistical comparison of isotype distribution of SARS-CoV-2-specific (top) and SARS-CoV-2 cross-reactive (bottom) B cells in the pre-vaccine and day 42 post-vaccination time points. The values in each table represent the number of antibody sequences with the designated isotypes. **D** Evolution of cross-reactive SARS-CoV-2 and SARS-CoV-2-only B cells from each isotype (separate plots) over time. Each line shows the number of B cells (*y* axis) for either SARS-CoV-2-only (blue) or SARS-CoV-2 cross-reactive with other coronaviruses (designated colors) B cells at the four timepoints (*x* axis). **E** Individual IgM, IgA, and IgG-expressing cells graphed for cumulative cross-reactive (MaxCOV) and SARS-CoV-2 LIBRA-seq scores shaded based on *k* = 4 nearest neighbors averaging for the time of sample collection. All error bars are mean ± standard deviation.

While numerous antigen-specific antibodies have been isolated from SARS-CoV-2 infection^39–41^ and more recently from vaccination^42^, little is currently known about the temporal trajectory of the antigen-specific antibody repertoire at single-cell resolution from SARS-CoV-2 vaccinees. We thus set out to characterize the evolution of the SARS-CoV-2-specific B-cell repertoire by analyzing multiple timepoints pre- and post-vaccination of a single healthy male Caucasian participant of age 45–50 (Fig. 1B) with LIBRA-seq^32,43^. Blood was also collected from this participant at Vanderbilt University Medical Center late in 2020 and early 2021 and the participant reported no prior history of SARS-CoV-2 infection. The antigen specificity of the polyclonal plasma from this participant was largely similar to what was observed for the ten-participant cohort described above, with BNT162b2 immunization leading to the emergence of SARS-CoV-2-reactive IgM, IgA, and IgG antibodies (Fig. 6A). While some reactivity with the closely related coronavirus SARS-CoV was observed, little reactivity was observed against the more distantly related coronavirus MERS-CoV. Although SARS-CoV-2 S-reactive IgA and IgG were present at day 14 post-vaccine prime, neutralizing antibody titers were first detected on day 28 (7 days post-boost) at levels that remained largely unchanged at day 42 (Fig. 6B).

LIBRA-seq enables high-throughput unbiased mapping of BCR sequence to antigen specificity for large numbers of B cells per sample against a diverse set of coronavirus antigens. Using a LIBRA-seq antigen library consisting of DNA oligo-tagged S proteins from SARS-CoV-2, SARS-CoV-2 D614G, SARS-CoV, MERS-CoV, HcoV-HKU1, HcoV-OC43, as well as the negative control antigens, B cells were enriched for antigen-positive cells by FACS (Supplementary Fig. 6A) and mapped to their antigen reactivity profile utilizing a next-generation sequencing readout. Clonal lineage tracing of BCR sequences from each timepoint identified several clusters containing sequences with high LIBRA-seq scores for SARS-CoV-2 (≥1) across multiple time points (Supplementary Fig. 6B). LIBRA-seq identified both B cells that were specific to SARS-CoV-2 (referred to as SARS-CoV-2-only B cells) as well as B cells that were cross-reactive between SARS-CoV-2 and other coronaviruses (cross-reactive B cells) (Supplementary Fig. 7). Both SARS-CoV-2-only and cross-reactive B cells showed a transition from IgM pre-vaccination to IgG after vaccination, suggesting antigen-driven class switching (Fig. 6C). IgM remained more prevalent than IgG in the cross-reactive B-cell population, suggesting a stronger class-switching potential for SARS-CoV-2-only over cross-reactive B cells in response to vaccination (Fig. 6C). SARS-CoV-2 cross-reactivity was primarily observed in HCoV-OC43-reactive IgM^+^ B cells in this participant. This was most predominant in the pre-vaccination and day 8 post-vaccination time points, with the overall levels of SARS-CoV-2 cross-reactive B cells generally decreasing at later timepoints post-vaccination, particularly among IgG^+^ B cells (Fig. 6D). The levels of SARS-CoV-2-only IgG^+^ B cells and IgG^+^ B cells that cross-reacted only between SARS-CoV-2 and the closely related SARS-CoV were substantially increased on days 14 and 42. Interestingly, while the levels of SARS-CoV-2-reactive B cells peaked at day 14 for both the IgG and IgA isotypes, the IgA levels were reduced to near baseline at day 42, while IgG levels at day 42 were decreased but clearly present. Changes in B-cell cross-reactivity and isotype abundance were also evident when analyzed at the single-cell level using antigen-specific LIBRA-seq scores, a metric for classifying antigen reactivity of B cells at single-cell resolution. When SARS-CoV-2 reactivity was compared to the combined LIBRA-seq scores for reactivity to all other coronavirus antigens measured over time, a trend was observed with cells skewing towards SARS-CoV-2-specific from more cross-reactive phenotypes at day 0 (Fig. 6E). These results suggest an evolution in SARS-CoV-2 antibody specificity and cross-reactivity in response to BNT162b2 vaccination, supporting a model of immune progression from the early prevalence of endemic cross-reactive IgM to more highly selective SARS-CoV-2 IgG-switched B cells over time.

### SARS-CoV-2-binding memory B cells and plasmablasts expand following BNT162b2 vaccination

**Fig. 7:**
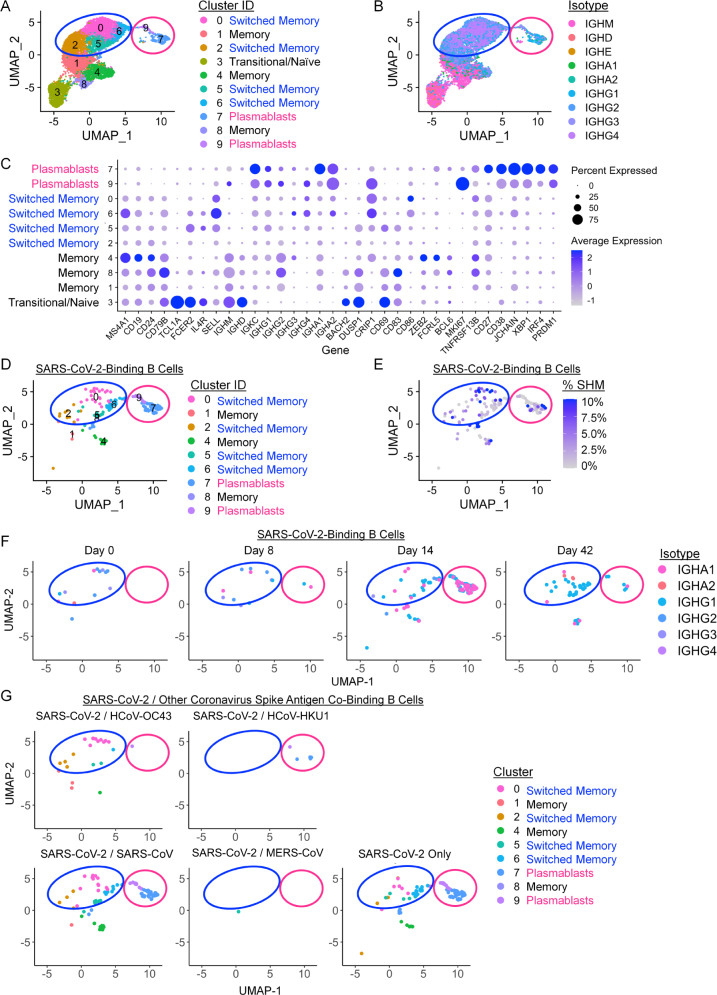
BNT162b2 vaccination drives IgG and IgA-switched SARS-CoV-2-binding memory B-cell and plasmablast expansion. B cells that bound a panel of LIBRA-seq antigens were purified from peripheral blood samples and single-cell RNA-seq/BCR-seq/LIBRA-seq data were integrated. Cells were scored positive for binding a given antigen for LIBRA-seq scores ≥1. **A** UMAP identified clusters of B cells based on transcriptional similarity. Antibody-secreting cells (pink circle encompassing clusters 7 and 9) and isotype-switched memory B cells (blue circle encompassing clusters 0, 2, 5, and 6) are highlighted across all UMAP plots. **B** BCR isotypes are shown among sorted B cells. **C** Selected gene expression profiles within each cluster are shown. **D**–**F** SARS-CoV-2-binding B cells were identified using LIBRA-seq and non-class-switched B cells were filtered out for detailed analysis. **D** UMAP identified clusters of SARS-CoV-2-binding B cells based on transcriptional similarity. **E** IgH somatic hypermutation frequency of SARS-CoV-2-binding B cells is indicated. **F** SARS-CoV-2-binding B-cell isotypes are shown for each time point. **G** SARS-CoV-2-binding B cells are shown, each panel indicates cells that also bound the indicated coronavirus S antigens.

The LIBRA-seq BCR sequence and antigen specificity data were further combined with RNA-seq data to identify gene expression patterns and transcriptionally define B-cell subset identities of SARS-CoV-2-binding B cells over time following vaccination. Nine clusters were identified by UMAP across all B cells based on gene expression profiles that represented naive, predominantly IgM memory (termed “memory”), predominantly IgA or IgG-switched memory (termed “switched memory”), or plasmablast populations (Fig. 7A, B, Supplementary Fig. 8A, B). Characteristic gene expression patterns defined each B-cell subset (Fig. 7C, Supplementary Fig. 8C). The analysis focused specifically on B cells predicted by LIBRA-seq to be SARS-CoV-2-binding showed only a modest number of differentially expressed genes compared to non-SARS-CoV-2-binding B cells (Supplementary Fig. 9A). These included, however, selectively elevated *CCR9*, *IgHM*, and *IGLC1* and decreased Galectin 1 (*LGALS1*) in the SARS-CoV-2-binding plasmablast populations, suggesting active migration and antibody production. Consistent with plasmablast populations identified by T-REX analysis of B-cell protein expression (Fig. 4), LIBRA-seq suggested SARS-CoV-2-binding B cells were represented among both switched memory and plasmablast cell clusters (Fig. 7D, Supplementary Fig. 9B). Analysis of class-switched, SARS-CoV-2-binding B cells found the majority had no or only modest degrees of somatic hypermutation, which did not increase with time (Fig. 7E, Supplementary Fig. 9C). By day 14 a robust SARS-CoV-2-binding plasmablast population was evident that diminished by day 42 after the initial BNT162b2 vaccination (Fig. 7F). These cells appeared to show an enrichment of class-switched plasmablasts on day 14 to switched memory cells on day 42 using a wide range of *IGHV* gene segments (Fig. 7F, Supplementary Fig. 9D). The plasmablast population did not have significant LIBRA-seq cross-reactivity scores for endemic coronaviruses but was selective for SARS-CoV-2 or with dual selectivity for SARS-CoV-2 and SARS-CoV (Fig. 7G).

### Integrated associations of antigen-specific cell and antibody responses to BNT162b2

**Fig. 8:**
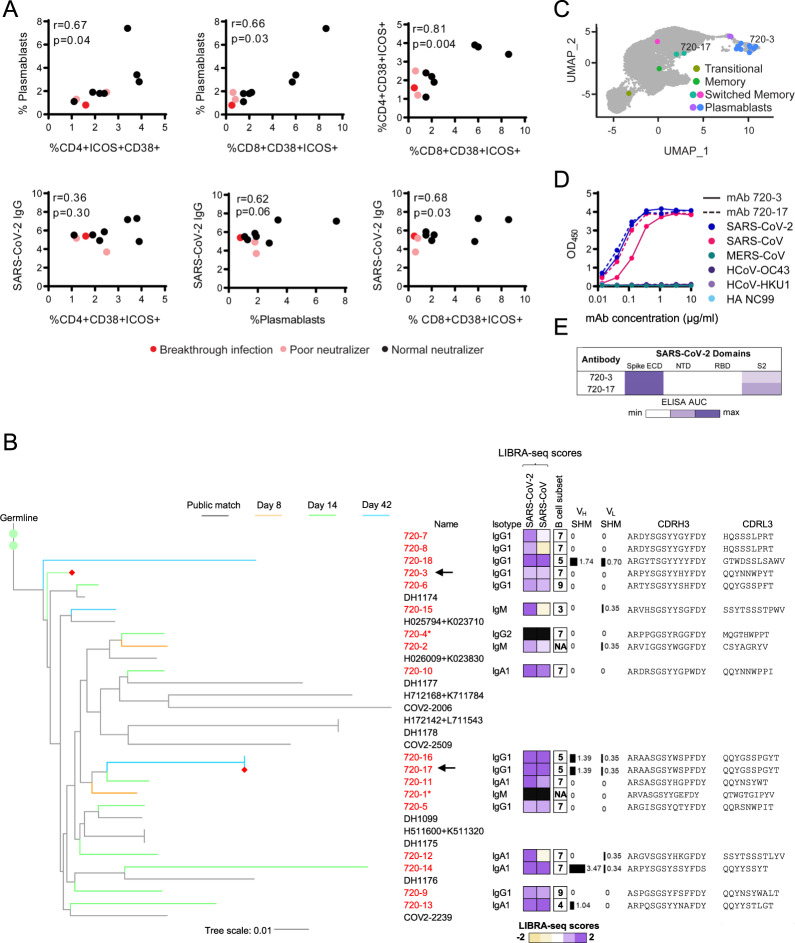
Associations of antigen-specific cell populations and antibody responses. **A** Correlation of biaxially gated plasmablast and T-cell populations with other cellular subsets from CyTOF analyses in Figs. 2 and 4 with SARS-CoV-2 IgG using Pearson correlations. Patients with typical neutralization titers are shown in black, poor neutralizers with no known breakthrough infection are shown in pink, and a poor neutralizer with known breakthrough infection is shown in red. **B** Phylogenetic tree of a public antibody cluster comprised of LIBRA-seq-identified sequences (antibody names: red; branches: colored by timepoint) and previously published SARS-CoV-2 antibody sequences from the CoV-AbDab database (gray), with recombinantly expressed antibodies 720-3 and 720-17 highlighted with a red diamond at the end of their respective branches and arrows. For each sequence from the cluster, sequence features including isotype, LIBRA-seq scores, % SHM of V-gene, and amino acid sequences of CDRH3 and CDRL3 are displayed. Nucleotide level percentage of SHM changes of V-genes of both heavy and light chains were reported as bars with numerical values. LIBRA-seq scores are shown as a range with tan-white-purple representing -2 to 0 to 2. Scores higher or lower than this range are shown as −2 and 2, respectively. The B-cell subsets are classified by scRNA-seq analysis. Sequences denoted by an asterisk (720-1 and 720-2) did not fulfill the cutoff for RNA-seq analysis and therefore were not classified into a B-cell subset. Antibodies 720-1 and 720-4 do not have a computed LIBRA-seq score due to not meeting data filtering criteria, as described in the methods. **C** Location of B cells in cluster 720 including recombinantly expressed monoclonal antibodies (720-3 and 720-17) on UMAP identified clusters of B cells based on transcriptional similarity. **D** ELISAs for antibodies selected for monoclonal antibody characterization are shown against spike proteins for SARS-CoV-2 (blue), SARS-CoV (pink), MERS-CoV (teal), HCoV-OC43 (purple), HCoV-HKU1 (light purple), and HA NC99 (light blue) for antibodies 720-3 (solid lines) and 720-17 (dashed lines). **E** Epitope mapping ELISA AUC for antibodies 720-3 and 720-17 against SARS-CoV-2 spike ECD, and truncated subunit domains NTD, RBD, and S2. ELISAs were performed in technical duplicate and repeated once.

To test associations of the antigen-specific CD4^+^, CD8^+^ T, and plasmablast populations and the antibody response to BNT162b2, the abundance of CD4^+^ICOS^+^CD38^+^ or CD8^+^ICOS^+^CD38^+^ and plasmablast cell populations were compared across participants (Fig. 8A). Pearson correlations identified statistically significant associations between the responding CD4^+^, CD8^+^, and plasmablast populations, supporting a coordinated model vaccine response. Further, plasmablast frequencies showed a trend towards significance, and CD8^+^ICOS^+^CD38^+^ T cells correlated with eventual levels of SARS-CoV-2-specific IgG. Although additional populations were identified by T-REX as selectively expanding after vaccination, no others were statistically significant relative to SARS-CoV-2 IgG titer. The three participants with the lowest levels of neutralizing antibodies (red and pink) also had the lowest numbers of CD8^+^ICOS^+^CD38^+^ T cells. Intriguingly, participant 2 (red) was subsequently infected with SARS-CoV-2 and had the lowest abundance of CD8^+^ICOS^+^CD38^+^ T cells and plasmablasts. While participant 2 represents a single case, the observation supports the idea that BNT162b2-induced expansion of memory CD4^+^ICOS^+^CD38^+^ and CD8^+^ICOS^+^CD38^+^ T cells may be critical to driving plasmablast expansion, antibody production, and protective immunity to SARS-CoV-2.

We next examined the relatedness at the sequence level of B cells elicited by vaccination. Interestingly, a cluster of antibody heavy chain sequences, referred to as cluster 720, was found to exhibit high similarity with public antibody sequences from the CoV-AbDab database^44^, a collection of antibody sequences from individuals with natural COVID-19 infection (Fig. 8B). Consistent with previous studies, this is indicative of a convergent B-cell response from natural infection and vaccination^45^. Interestingly, however, despite the high similarity in heavy chain sequences, the members of cluster 720 utilized a diversity of light chain V-genes and CDRL3 sequences. The B cells from cluster 720 included sequences with IgM, IgA, and IgG isotypes, and originated from switched memory and plasmablast cell clusters (Fig. 8C) that displayed only low to moderate degrees of somatic hypermutation (Fig. 8B). Day 8 sequences from cluster 720 were of IgM isotype, whereas the sequences from days 14 and 42 appeared as IgG and IgA isotypes (Fig. 8B). To characterize individual antibodies from cluster 720, we expressed heavy- and light- chain pairs as recombinant IgG for two members from diverse branches in the phylogenetic tree, 720-3 (using IGKV3-15) and 720-17 (using IGKV3-20), and tested for reactivity against coronavirus S proteins. Consistent with their LIBRA-seq scores, these antibodies were cross-reactive to SARS-CoV-2 and the closely related SARS-CoV but showed no reactivity to the endemic coronaviruses HCoV-HKU1 and HCoV-OC43 (Fig. 8D). Epitope mapping experiments demonstrated that these two antibodies were specific to the S2 domain of the spike (Fig. 8E, Supplementary Fig. 10).

## Discussion

Here we used unbiased approaches to describe the antigen-specific cellular response and development of the specific antibody repertoire over time in an in-depth analysis of a focused longitudinal cohort of healthy recipients of the BNT162b2 vaccine. While participants were reported to be healthy and without prior SARS-CoV-2 infection and our serology showed a lack of neutralizing antibodies, we cannot formally exclude undetected prior asymptomatic infection. Our findings are focused to provide a detailed analysis of one specific RNA-based vaccine, and it will be important to compare to other SARS-CoV-2 vaccine platforms. Machine learning analyses of proteomic mass cytometry data and single-cell sequencing of the SARS-CoV-2 S B-cell repertoire here identified expanding antigen-specific CD4^+^ T-cell, CD8^+^ T-cell, B-cell, and plasmablast populations. The B-cell repertoire shifted from initial apparent cross-reactivity with endemic coronavirus prior to immunization to a more SARS-CoV-2-selective response later, marked by the expansion of IgA and IgG plasmablasts. Importantly, antigen-specific cell subsets identified early after vaccination correlated with a later sustained IgG antibody response at day 105. Notably, both the CD4^+^ and CD8^+^ populations of ICOS^+^CD38^+^ T cells were deficient in a participant who later experienced a breakthrough SARS-CoV-2 infection between days 105–180.

Expanding SARS-CoV-2 S protein-specific CD4^+^ICOS^+^CD38^+^ cells identified through unbiased machine learning resembled CD38^+^ICOS^+^CXCR5^−^ T cells that were previously identified following SARS-CoV-2 infection^4^ and a prior study of BNT162b2 vaccination^46^, but had several previously undescribed characteristics. A subset of these antigen-specific cells expressed BCL6, although expression of this Tfh-associated transcription factor was decreased in this subset after boost. PD1^+^ICOS^+^ cTfh cells have been previously associated with vaccine responses^47–49^ and expansion of cTfh was observed after BNT162b2 vaccination^46^. However, T cells identified by T-REX expressed little or no CXCR5 which is characteristic of Tfh and cTfh. The ICOS^+^CD38^+^ antigen-specific population observed here may resemble rhinovirus-specific tissue homing memory CD4^+^ T cells that express CCR5 and CD38 and are associated with transcription factor TBET^50^ and extrafollicular CD4^+^PD-1^+^CXCR5^−^ T cells observed following viral infections^51^. Similarly, expanding CD4^+^ICOS^+^CD38^+^ and CD8^+^ICOS^+^CD38^+^ T-cell populations share features of pathogenic Tph cells that were previously described in the synovium of rheumatoid arthritis patients that were CXCR5^−^PD1^+^BCL6^lo^ and retained a potent ability to promote B-cell differentiation^35^. Expanding T cells following BNT162b immunization thus appear as antigen-experienced memory Tfh-like or Tph T cells that may be poised for tissue homing to support long-lived anti-viral memory responses.

In addition to induction of SARS-CoV-2 neutralizing antibodies^52^, cytotoxic T-cell responses also contribute to vaccine-mediated protection. CD8^+^ T cells support life-long immunity against influenza^53^, EBV^54^, and CMV^55^, and induction of a robust CD8^+^ T-cell response is an emerging focus in vaccine development^56,57^. Here, we identified an antigen-specific CD8^+^ICOS^+^CD38^+^ cytotoxic memory T-cell population capable of cytokine production in response to S1 antigenic peptides, although Granzyme B was low, suggesting reduced cytotoxicity. Despite a lack of CXCR5 expression, a subset of these cells expressed BCL6 and they share features with CD8^+^ Tfh that have been described as effectors in patients with chronic hepatitis B infection^58^. In addition to CD8^+^ T-cell-mediated cellular immunity, this CD8^+^ T-cell subset was most associated with plasmablasts and SARS-CoV-2-specific IgG. Interestingly, the participant with breakthrough SARS-CoV-2 infection failed to generate CD8^+^CD38^+^ICOS^+^ T cells. If confirmed in a larger cohort, this CD8^+^CD38^+^ICOS^+^ T-cell subset may provide a marker of successful immunization and protection following SARS-CoV-2 vaccination. Such a biomarker may be critical as additional variants of concern emerge as S-specific CD4^+^ T-cell responses appear to be less impacted by variants of a concern than humoral immunity^59,60^.

SARS-CoV-2 S protein-specific B cells identified here exhibited similar phenotypic properties as the previous studies^61^ and our findings suggest a modest contribution of cross-reactivity with endemic coronaviruses. Plasmablasts had high expression of CD71, an iron transport receptor that is commonly expressed on antigen-specific B-cell subsets including in the context of vaccination^62,63^, and proteins for mitochondrial amino acid and lipid metabolism. Reports of CD71 expression after COVID-19 infection^64,65^ are consistent with an overlap of B-cell activation across mRNA vaccination and natural SARS-CoV-2 infection. Limited pre-existing immunity to endemic coronaviruses prior to SARS-CoV-2 vaccination was detected in this study, albeit at varying levels between participants. Although a previous report noted an increased serological response in recovered COVID-19 individuals^66^, we noted only modest changes in HCoV-OC43 serology following vaccination. Instead, LIBRA-seq antigen specificity data showed an initial HCoV-OC43, SARS-CoV-2 cross-reactive IgM-expressing B-cell response that decreased in frequency over time and evolved to IgA- and IgG-switched B cells with greater SARS-CoV-2 specificity. Given HCoV-OC43 and HCoV-HKU1 utilize different cell receptors for host entry, the lack of correlation between pre-existing coronavirus immunity and SARS-CoV-2 neutralization is not surprising. Instead, antibodies that target the more conserved S2 portion of the spike may promote anti-viral function via Fc effector function mechanisms. Indeed, pre-existing immunity to the endemic coronavirus HCoV-OC43 has been previously reported to associate with survival outcomes from COVID-19 infection^67^. T cells were also previously reported to share cross-reactivity with endemic coronaviruses, although most SARS-CoV-2 clonally expanded T cells are derived from naive populations^68^. Further investigation into this potential association is needed, given an independent study reported no correlation between endemic coronavirus immunity and survival after COVID-19 infection^66^.

The longitudinal characterization of the B-cell repertoire of a BNT162b2-vaccinated participant found the presence of a cluster of public antibody heavy chain sequences with high similarity to antibody sequences observed in natural infection. These public heavy chain sequences were observed at multiple time points after vaccination, with IgM members identified on day 8, and IgG/IgA sequences identified at later time points, suggesting antigen-driven evolution of a public mode of SARS-CoV-2 spike recognition. Interestingly, despite the high similarity in heavy chain sequences, antibodies from this public cluster utilized a variety of different light chain V-genes and CDRL3 sequences. This suggests that the immune response to SARS-CoV-2 may utilize a combination of common heavy chain sequence characteristics that are important for antigen recognition, paired with a heterogeneous set of light chain sequences that may allow for additional diversification of the fine epitope specificities of responding B cells. The ability to increase the diversity in B-cell responses, while retaining critical antigen interactions, may be an important tool that the human immune system utilizes for counteracting virus evasion mechanisms.

Immunization with BNT162b2 in the participant cohort also resulted in a transient SARS-CoV-2 IgA response. We were unable to determine here if this was monomeric or dimeric IgA based on our serology. The polysaccharide pneumococcal vaccine can also lead to an IgA vaccine response^69^, although robust IgA responses are not generated by either the intramuscular influenza^70^ or tetanus-diphtheria-acellular pertussis vaccine^71^. IgA is critical in the SARS-CoV-2 early neutralizing antibody response^72^ and was previously observed together with IgG in response to the SARS-CoV-2 mRNA vaccine^73–75^. Consistent with the development of protective immunity, antigen-binding B cells were IgG and IgA class-switched and primarily observed among memory B-cell and plasmablast subsets. The strong IgA response elicited by the SARS-CoV-2 mRNA vaccines may arise as a consequence of single-stranded RNA directly stimulating B cells^76,77^ or activation of Toll-like receptor 7 (TLR7) on dendritic cells^78^. Indeed, the addition of a single-stranded RNA adjuvant to a traditional influenza vaccine generated mucosal immunity through a robust IgA response and provided more strain cross-protection^79^.

This study uses unbiased machine learning and sequencing technologies to identify the antigen-specific cells and evolving antibody response to RNA-based vaccination against SARS-CoV-2. While the limited sample size and focus on specific timepoints with only one vaccine platform prevents broad population-based or time-dependent conclusions, our unbiased approach allowed isolation of live, virus-specific T cells and identified features of the antigen-specific B-cell response that will facilitate further studies and development of additional therapies. The multi-compartment vaccine response induced by BNT162b2 may have important ramifications, particularly in immunocompromised populations. Reduced humoral responses have been reported to SARS-CoV-2 infection or BNT162b2 in patients with solid organ transplant^26,80–84^, cancer^85,86^, hemodialysis^26^, or immune-mediated inflammatory diseases^87,88^. The identification of altered proteins or specific cell populations to serve as biomarkers for successful vaccination may provide important insight to monitor the development and continued protection of these vulnerable populations. While limited sample sizes here reduce statistical power for population-level correlations and detailed outcomes, the unbiased identification of antigen-specific CD4^+^ T-cell, CD8^+^ T-cell, and B-cell populations provides important insight into general mechanisms of RNA-based vaccines and the cellular basis for vaccine-induced antibodies and protection from SARS-CoV-2.

## Methods

### Human participant information

All research in this study complies with relevant ethical regulations. Vanderbilt University Medical Center Institutional Review Board approval was obtained (VUMC 191562). Participants were recruited via email listserv in December 2020 in the United States. Informed consent was obtained, and a baseline health questionnaire including knowledge of prior SARS-CoV-2 infection was also completed. Participants were offered an Amazon gift card as compensation for their time.

### Study design, statistics, and reproducibility

No statistical method was used to predetermine sample size. Simple phlebotomy was performed either pre-vaccine (day 0), day 28–30, and day 95–100 OR pre-vaccine and on days 8, 14, and 42 after initial BNT162b2 vaccination using sodium citrate mononuclear cell preparation (CPT) tubes (BD, 362761). All participants received two doses of vaccine 21 days apart. A survey regarding COVID breakthrough infections was completed on day 180. No data were excluded from the analysis, and the experiments were not randomized. Investigators were blinded to the presence of breakthrough infections until all data analysis was complete. Research assays performed on human samples were conducted as a single batch according to best practices in the field using a pair of samples from *N* = 10 independent individuals run on multiple assays that included both redundant and complementary tests (i.e., CyTOF T-cell panel, CyTOF B-cell panel, and fluorescence flow cytometry). While sample availability limited most assays to be run as a single batch, antibodies overlap between panels allowing for some antigens and cell types to be measured two or three times. Specific statistical tests are described within each figure and method. All relevant code is published and available through indicated references.

### PBMC and plasma isolation

CPT tubes were spun at 1600 RCF for 20 min. The plasma layer was carefully removed, transferred to a conical vial, spun at 600 × *g* for 10 min, and the supernatant transferred to microtubes in 1 mL aliquots. Plasma was stored at −80 °C until further use. Buffy coat was divided among two clean conical tubes. CPT tubes were rinsed with 1 mL PBS (ThermoFisher, 10010-049), and the total volume in the conical was increased to 15 mL Cells were pelleted at 600 × *g* for 10 min. Cell pellets were combined and washed with 10cc PBS, and then cells were pelleted again at 600 × *g* for 10 min. PBS was discarded, and the pellet was resuspended in 3 mL ACK buffer (ThermoFisher, A1049201) for 5 min. In all, 10 mL of PBS was added to the ACK cell suspension, and cells were pelleted for 10 min at 600 × *g*. Cells were resuspended in PBS, strained through a cell strainer (Falcon, 352235), and counted using an ACT Diff hematology analyzer (Beckman Coulter). Cells were pelleted by centrifugation at 600 × *g* for 10 min and resuspended in heat-inactivated FBS (GemCell, 100–500) containing 10% DMSO (Sigma-Aldrich, D2650) at a concentration of 5 million cells/mL in cryovials (ThermoFisher, 5000–0020). Cryovials were frozen overnight to −80 °C using Mr. Frosty freezing containers (ThermoFisher, 5100–0050) and then transferred to liquid nitrogen for long-term storage.

### Mass cytometry PBMC staining

Metal-tagged antibodies were purchased from Fluidigm. Cell labeling and mass cytometry analysis were performed as previously described^89,90^. Briefly, cells were incubated with a viability reagent (Cell ID Intercalator-Rh; Fluidigm), per the product literature. Then cells were washed in PBS without calcium or magnesium (Gibco, Thermo Fisher Scientific) containing 1% BSA (Thermo Fisher Scientific) and stained in 50 μL PBS and BSA 1%–containing antibody cocktail for extracellular targets. Cells were stained for 30 min at room temperature using the antibodies listed in Supplementary Table 1. Cells were washed in PBS and BSA 1% and then fixed with 1.6% paraformaldehyde (Electron Microscopy Sciences). Cells were washed once in PBS and permeabilized by resuspension in ice-cold methanol. After incubation overnight at −20 °C, cells were washed with PBS and BSA 1% and stained in 50 μL PBS and BSA 1%–containing antibody cocktail for intracellular targets. Cells were washed in PBS and BSA 1%, then washed with PBS and stained with an iridium DNA intercalator (Fluidigm) for 20 min at room temperature. Finally, cells were washed with PBS and with diH_2_O before being resuspended in 1× EQ Four Element Calibration Beads (Fluidigm) and collected on a Helios mass cytometer (Fluidigm) at the Vanderbilt Flow Cytometry Shared Resource Center. Events were normalized as previously described^91^.

### Mass cytometry data analysis

After normalization, CYTOF data were scaled with an arcsinh transformation, with an appropriate cofactor set for each channel following standard procedures for fluorescence and mass cytometry data^92^. Data were then manually gated for removal of atypical events^93^. After quality control gating, a UMAP analysis was performed on the cleaned-up samples using the surface markers in each panel. Metabolic markers, ki67, iridium, and rhodium were not used to create the UMAP. The resulting common, two-dimensional embedding of the data was used for visualization and selection of either CD3^+^ T cells (from T-cell panel data) or B cells (from B-cell panel data) for further downstream analysis. A common t-SNE analysis was done on all 10 participant samples using the same markers used to create the UMAP on either the CD3^+^ T cells or B cells extracted from their respective UMAP. After the t-SNE, equally sampling was done on each participant pair. The 10 participant samples were then combined for a T-REX comparison of day 0 and day 28 data^94^. MEM was used to quantify enriched features in each region of significant change^33^.

### Mass cytometry statistical analysis

Comparisons of population frequencies pre- and post-vaccination as well as correlations between post-vaccine cell frequencies and IgG titers were done in GraphPad Prism version 9.0. Populations were compared using Mann-Whitney U tests. Statistical correlations were determined using Pearson correlations. *P* values < 0.05 were considered statistically significant.

### In vitro characterization of CD38^+^ICOS^+^ Cells and quantification of CD38^+^ICOS^+^ CD4^+^ and CD8^+^ T cells by flow cytometry

To quantify CD4^+^ICOS^+^CD38^+^ and CD8^+^ICOS^+^CD38^+^ T cells, PBMCs were first resuspended with Human TruStain Fcx (Biolegend) for 10 min at room temperature and then stained with the following antibodies in FACS buffer (PBS + 2% fetal bovine serum): CD8a e450 (Invitrogen 48-0086-42, 1:200) ICOS BV605 (Biolegend 313538, 1:50), CCR7 PE (Biolegend 353204, 1:200), CD38 PerCP (Biolegend 303520, 1:100), CD4 PECy7 (Biolegend 357410, 1:100), and CD3 APCCy7 (Biolegend 300318, 1:200). Tfh markers consisted of CXCR5 APC (Biolegend 356907, 1:200) and PD-1 PE (Biolegend 135205, 1:100). CD71 APC (Biolegend 334108, 1:100) was also measured on sorted cells. Cells were analyzed on a Miltenyi MACSQuant16 Analyzer with single-stain control PBMC samples used for compensation conducted in FlowJo v10.6.2.

### Stimulation of ICOS^+^CD38^+^ and ICOS^lo^CD38^lo^ populations with SARS-CoV-2 S protein

The same staining procedure was used for FACS of ICOS^+^CD38^+^ and ICOS^lo^CD38^lo^ populations on a BD FACSAria III instrument in the Vanderbilt University Medical Center Flow Cytometry Shared Resource Core. Sorted cells were stained with 1 μM CellTrace Violet (CTV, ThermoFisher, c34557) for 20 min and then cultured with autologous unlabeled PBMC samples from day 0 (pre-vaccination) samples. Before adding CTV-labeled cells to PBMCs for activation, PBMCs were CD3-depleted by positive selection (CD3 Microbeads, Miltenyi Biotec, 130-050-101) and purity of CD3 depletions were assessed by flow cytometry. Cells were cultured in Human Plasma-like Media (HPLM, Thermofisher, A4899102)^95^ + 1% pen/strep + 5% dialyzed serum (Sigma-Aldrich, F0392). For SARS-CoV-2 peptide stimulation, cultures were treated with Peptivator SARS-CoV-2 Prot_S peptide pool (Miltenyi Biotec, 130-126-700) in a 48-well plate format for 2 days.

For cytokine staining, cells were either stimulated with PMA (1μg/ml) and ionomycin (750 ng/ml) for 5 h or restimulated on day 11 in 96-well plates with 2.5 μg/ml recombinant SARS-CoV-2 Spike protein S1 (Biolegend 792906, carrier-free) for 8 h in the presence of 1 μg/ml Golgiplug and 0.7 μg/ml Golgistop. Peptivator-stimulated cultures were treated with Golgiplug/Golgistop overnight after 2 days of activation. Cells were surface-stained in FACS buffer, fixed with 1.5% paraformaldehyde for 10 min, and permeabilized with methanol for 20 min on ice. Additional intracellular antibodies were: TNF AF488 (Biolegend 502915, 1:100), IFN-γ APC (Invitrogen 17-7319-82, 1:100) IL-2 AlexaFluor 700 (Biolegend 500320, 1:150), granzyme b FITC (Biolegend 515403, 1:100), phospho-S6 APC Ser235/236 (Invitrogen 17-9007-42, 1:80), Bcl6 FITC (Biolegend 358513, 1:100), and Glut-1 AlexaFluor 647 (Abcam ab115730, 1:300). Cytokines measured from PBMC supernatants were collected after 4 days of incubation with S protein and concentrations were predicted using a standard curve in the LEGENDplex assay (Miltenyi Biotec 741028).

### In vitro stimulation statistical analysis

Group comparisons were performed using GraphPad Prism version 9.0. Populations were compared using Mann-Whitney U tests. P values less than 0.05 were considered statistically significant.

### Recombinant expression and purification of coronavirus antigens

Plasmids encoding residues 1–1208 of the SARS-CoV-2 spike with a mutated S1/S2 cleavage site, proline substitutions at positions 817, 892, 899, 942, 986 and 987, and a C-terminal T4-fibritin trimerization motif, an 8x HisTag, and a TwinStrepTag (SARS-CoV-2 S HP); 1–1208 of the SARS-CoV-2 spike with a mutated S1/S2 cleavage site, proline substitutions at positions 817, 892, 899, 942, 986 and 987, as well as mutations L18F, D80A, L242-244L del, R246I, K417N, E484K, N501Y, and a C-terminal T4-fibritin trimerization motif, an 8x HisTag, and a TwinStrepTag (SARS-CoV-2 S HP Beta); 1–1208 of the SARS-CoV-2 spike with a mutated S1/S2 cleavage site, proline substitutions at positions 817, 892, 899, 942, 986, and 987, as well as mutations 69-70del, Y144del, N501Y, A570D, P681H, and a C-terminal T4-fibritin trimerization motif, an 8x HisTag, and a TwinStrepTag (SARS-CoV-2 S HP Alpha); residues 1-1190 of the SARS-CoV spike with proline substitutions at positions 968 and 969, and a C-terminal T4-fibritin trimerization motif, an 8x HisTag, and a TwinStrepTag (SARS-CoV S-2P); residues 1-1291 of the MERS-CoV spike with a mutated S1/S2 cleavage site, proline substitutions at positions 1060 and 1061, and a C-terminal T4-fibritin trimerization motif, an AviTag, an 8x HisTag, and a TwinStrepTag (MERS-CoV S-2P Avi); residues 1–1277 of the HCoV-HKU1 spike with a mutated S1/S2 cleavage site, proline substitutions at positions 1067 and 1068, and a C-terminal T4-fibritin trimerization motif, an 8x HisTag, and a TwinStrepTag (HCoV-HKU1 S-2P); residues 1-1278 of the HCoV-OC43 spike with proline substitutions at positions 1070 and 1071, and a C-terminal T4-fibritin trimerization motif, an 8x HisTag, and a TwinStrepTag (HCoV-OC43 S-2P); were transiently transfected into FreeStyle293F cells (Thermo Fisher) using polyethylenimine. For all antigens with the exception of SARS-CoV-2 S HP, cells were treated with 1 µM kifunensine to ensure uniform glycosylation three hours post-transfection. Transfected supernatants were harvested after five days of expression. SARS-CoV-2 S HP, SARS-CoV S-2P, MERS-CoV S-2P, HCoV-HKU1 S-2P, and HCoV-OC43 S-2P were purified using StrepTrap columns. SARS-CoV-2 S HP, SARS-CoV-2 S HP Beta, SARS-CoV-2 S HP Alpha, SARS-CoV S-2P, MERS-CoV S-2P, HCoV-HKU1 S-2P, and HCoV-OC43 S-2P were purified over a Superose6 Increase column (GE Life Sciences).

### Recombinant expression and purification of ZM197 Env and NC99 Hemagglutinin

Recombinant, soluble HIV-1 gp140 SOSIP trimer from strain ZM197 (clade) containing an AviTag and recombinant NC99 HA protein consisting of the HA ectodomain with a point mutation at the sialic acid-binding site (Y98F) to abolish non-specific interactions, a T4-fibritin foldon trimerization domain, AviTag, and hexahistidine-tag, were expressed in Expi 293 F cells using polyethylenimine transfection reagent and cultured. FreeStyle F17 expression medium supplemented with pluronic acid and glutamine was used. The cells were cultured at 37 °C with 8% CO_2_ saturation and shaking. After 5–7 days, cultures were centrifuged, and the supernatant was filtered and run over an affinity column of agarose-bound Galanthus nivalis lectin. The column was washed with PBS and antigens were eluted with 30 mL of 1 M methyl-a-D-mannopyranoside. Protein elutions were buffer exchanged into PBS, concentrated, and run on a Superdex 200 Increase 10/300 GL Sizing column on the AKTA FPLC system.

### Biotinylation of antigens

Constructs containing an Avitag (ZM197 Env and HA NC99) were biotinylated using the site-specific biotinylation kit according to manufacturer instructions (Avidity LLC.) All other antigens not containing an Avitag were non-specifically biotinylated using the EZ-Link Sulfo-NHS-Biotin kit at a 50:1 biotin:protein molar ratio.

### DNA-barcoding of antigens

We used oligos that possess 15 bp antigen barcode, a sequence capable of annealing to the template switch oligo that is part of the 10X bead-delivered oligos, and contain truncated TruSeq small RNA read 1 sequence in the following structure: 5’-CCTTGGCACCCGAGAATTCCANNNNNNNNNNNNNCCCATATAAGA*A*A-3’, where Ns represent the antigen barcode. We used the following antigen barcodes: GCAGCGTATAAGTCA (SARS-CoV-2 S HP), GACAAGTGATCTGCA (SARS-CoV-2 S HP D614G), GCTCCTTTACACGTA (SARS-CoV S), GGTAGCCCTAGAGTA (MERS-CoV S), TGTGTATTCCCTTGT (HCoV-HKU1 S), AGACTAATAGCTGAC (HCoV-OC43 S), TCATTTCCTCCGATT (ZM197 EnV), CTTCACTCTGTCAGG (HA NC99), Oligos were ordered from IDT with a 5’ amino modification and HPLC purified.

For each antigen, a unique DNA barcode was directly conjugated to the antigen itself. In particular, 5’amino-oligonucleotides were conjugated directly to each antigen using the Solulink Protein-Oligonucleotide Conjugation Kit (TriLink cat no. S-9011) according to the manufacturer’s instructions. Briefly, the oligo and protein were desalted, and then the amino-oligo was modified with the 4FB crosslinker, and the biotinylated antigen protein was modified with S-HyNic. Then, the 4FB-oligo and the HyNic-antigen were mixed together. This causes a stable bond to form between the protein and the oligonucleotide. The concentration of the antigen-oligo conjugates was determined by a BCA assay, and the HyNic molar substitution ratio of the antigen-oligo conjugates was analyzed using the NanoDrop according to the Solulink protocol guidelines. AKTA FPLC was used to remove excess oligonucleotide from the protein-oligo conjugates, which were also verified using SDS-PAGE with a silver stain. Antigen-oligo conjugates were also used in flow cytometry titration experiments.

### Antigen-specific B-cell sorting

Cells were stained and mixed with DNA-barcoded antigens and other antibodies, and then sorted using FACS. First, cells were counted and viability was assessed using Trypan Blue. Then, cells were washed three times with DPBS supplemented with 0.1% bovine serum albumin (BSA). Cells were resuspended in DPBS-BSA and stained with cell markers including viability dye (Ghost Red 780, Tonbo Biosciences, 13–0865, 1 µL per 10e6 total cells), CD14-APC-Cy7 (BD, 561709, 1 µL per 60e6 total cells), CD3-FITC (Tonbo Bioscience, 35–0037, 1 µL per 20e6 total cells), CD19-BV711 (BD, 563036, 1 µL per 10e6 total cells), and IgG-PE-Cy5 (BD, 551497, 3 µL per 10e6 total cells). Additionally, antigen-oligo conjugates were added to the stain (1 µg of every antigen except for HA NC99 and HCoV-HKU1 S which were added at 0.1 µg). After staining in the dark for 30 min at room temperature, cells were washed three times with DPBS-BSA at 300 g for five min. Cells were then incubated for 15 min at room temperature with Streptavidin-PE (Invitrogen, S886, 1 µL per 100e6 total cells) to label cells with bound antigen. Cells were washed three times with DPBS-BSA, resuspended in DPBS, and sorted by FACS. Antigen-positive cells were bulk sorted and delivered to the Vanderbilt Technologies for Advanced Genomics (VANTAGE) sequencing core at an appropriate target concentration for 10X Genomics library preparation and subsequent sequencing. FACS data were analyzed using FlowJo.

### Sample preparation, library preparation, and sequencing

Single-cell suspensions were loaded onto the Chromium Controller microfluidics device (10X Genomics) and processed using the B-cell Single Cell V(D)J solution according to the manufacturer’s suggestions for a target capture of 10,000 B cells per 1/8 10X cassette, with minor modifications in order to intercept, amplify and purify the antigen barcode libraries as previously described^32^.

### Sequence processing and bioinformatic analysis

We utilized our previously described pipeline to use paired-end FASTQ files of oligo libraries as input, process and annotate reads for cell barcode, UMI, and antigen barcode, and generate a cell barcode - antigen barcode UMI count matrix^43^. BCR contigs were processed using Cell Ranger v5.0.0 (10X Genomics) using GRCh38 as reference. Antigen barcode libraries were also processed using Cell Ranger (10X Genomics). The overlapping cell barcodes between the two libraries were used as the basis of the subsequent analysis. We removed cell barcodes that had only non-functional heavy chain sequences as well as cells with multiple functional heavy chain sequences. Additionally, we aligned the BCR contigs (filtered_contigs.fasta file output by Cell Ranger, 10X Genomics) to IMGT reference genes using HighV-Quest^96^. The output of HighV-Quest was parsed using Change-O^97^. and merged with an antigen barcode UMI count matrix. Finally, we determined the LIBRA-seq score for each antigen in the library for every cell as previously described^32^.

### LIBRA-seq data quality control filtering

Cells were filtered based on multiple criteria for further analysis. Cells were only included if the sum of all antigen UMI counts for a particular cell barcode was greater than 4. All cells that met these criteria from pre, day 8, day 14, and day 42 time points were combined. Then, we removed cells from the dataset that had multiple heavy chains or multiple light chains associated with a single-cell barcode. Then, LIBRA-seq scores were generated (8). Briefly, a pseudocount of 1 was added to each antigen UMI count, and then centered-log ratios (CLR) were calculated for each antigen UMI count for each cell. Then, an antigen-wise z-score transformation was applied. After performing the LIBRA-seq score calculation, cells were filtered out if they fulfilled any of the following criteria: (1) Max UMI among all antigens less than or equal to 30, (2) ZM197 UMI counts greater than or equal to 30, (3) HA UMI counts greater than or equal to 30, (4) MERS UMI counts greater than 10 times the max UMI of other CoV in that cell, or (5) 10 times ZM197 UMI counts greater than the max UMI of non-MERS-CoV in that cell. Supplementary Fig. 6B and Fig. 7B were generated using the pre-filtered data. Supplementary Fig. 7 and Fig. 5C–E used post-filtered data. Fig. 8B uses post-filtered data with the exception of antibody sequences 720-1 and 720-4 which did not meet filtering criteria, and thus do not have a computed LIBRA-seq score.

### Antibody sequence clustering

Using pre-filtered data, cells from multiple timepoints were combined together to identify highly similar antibody sequences between timepoints. Single-linkage clustering was performed using Change-O^97^ with the criteria of same VH- and JH-gene usage, same junction, and CDRH3 length, and 80% CDRH3 nucleotide sequence identity.

### Phylogenetic tree

For the selected cluster 720, phylogenetic analysis was performed to understand the heavy chain sequence similarities. The sequences from cluster 720 used IGHV3-30-3 and IGHJ4 genes and included 18 members from three different post-vaccination time points. The cluster 720 heavy chain sequences were compared to sequences from the Coronavirus antibody database (CoV-AbDab) to identify antibodies with high sequence similarity (based on the same VH-and JH-gene usage and 70% CDRH3 amino acid sequence identity) to at least one member of cluster 720. This resulted in the identification of 14 antibody sequences that fulfilled the similarity criteria with the cluster 720 sequences. To generate a phylogenetic tree of these public sequences, the tree was rooted in a putative germline sequence that was generated using the IGHV3-30-3 gene, a consensus CDRH3 sequence based on the cluster 720 members with 100% germline gene identity, and the IGHJ4 J-gene for framework 4. The tree was visualized using the Dendroscope tool^98^.

### Plasma serology ELISAs

In all, 100 µL of antigen was plated at a concentration of 500 ng/µL in PBS overnight at 4 °C. The following day plates are washed with 1× PBS and 0.1% Tween-20 (PBS-T) and then blocked with 5% non-fat dry milk (NFDM). After an hour of blocking at 1 h at RT, plates are washed 3× with PBS-T, and plasma is diluted in 1% NFDM PBS-T at a top concentration of 1:67 followed by 7 3-fold dilutions. The plates were incubated at RT for 1 h and then washed three times in PBS-T. The secondary antibody was added in 1% NFDM in PBS-T to the plates, which were incubated for one hour at RT. Plates were washed three times with PBS-T and then developed by adding TMB substrate to each well. The plates were incubated at room temperature for ten minutes, and then 1 N sulfuric acid was added to stop the reaction. Plates were read at 450 nm.

### Plasma VSV SARS-CoV-2 neutralization assay

In brief, 100 μL of plasma samples are heat-inactivated at 570 °C for 1 h, and starting at 1:25 dilution eight twofold serial dilutions were made with DMEM supplemented with 2% FBS. To determine the neutralizing activity of plasma/serum, we used a real-time cell analysis (RTCA) assay on an xCELLigence RTCA MP Analyzer (ACEA Biosciences Inc.) that measures virus-induced cytopathic effect (CPE). Briefly, 50 μL of cell culture medium (DMEM supplemented with 2% FBS) was added to each well of a 96-well E-plate using a ViaFlo384 liquid handler (Integra Biosciences) to obtain background reading. A suspension of 18,000 Vero cells in 50 μL of cell culture medium was seeded in each well, and the plate was placed on the analyzer. Measurements were taken automatically every 15 min, and the sensograms were visualized using RTCA software version 2.1.0 (ACEA Biosciences Inc). VSV SARS-CoV-2 (0.01 MOI, ~120 PFU per well) was mixed 1:1 with a dilution of plasma/serum in a total volume of 100 μL using DMEM supplemented with 2% FBS as a diluent and incubated for 1 h at 37 °C in 5% CO_2_. At 16 h after seeding the cells, the virus-plasma/serum mixtures were added in replicates to the cells in 96-well E-plates. Triplicate wells containing virus only (maximal CPE in the absence of mAb) and wells containing only Vero cells in medium (no-CPE wells) were included as controls. Plates were measured continuously (every 15 min) for 48 h to assess virus neutralization. Normalized cellular index (CI) values at the endpoint (48 h after incubation with the virus) were determined using the RTCA software version 2.1.0 (ACEA Biosciences Inc.). Results are expressed as percent neutralization in a presence of respective plasma/serum relative to control wells with no CPE minus CI values from control wells with maximum CPE. RTCA IC50 values were determined by nonlinear regression analysis using Prism software.

### Single-cell RNA-seq analysis

Single-cell analysis was performed using Seurat v4.0.0^99^. Cells with fewer than 200 RNA features that contained >10% mitochondrial genes were removed. Immunoglobulin VH, Vκ, and Vλ genes were removed prior to UMAP clustering of RNA-seq data to prevent them from driving transcriptionally-defined clusters. LIBRA-seq scores were used to identify SARS-CoV-2-binding B cells. B-cell subset identities were assigned to clusters based on transcriptional profiles that were consistent with other studies defining these populations. The Seurat FindMarkers function, which uses a non-parametric Wilcoxon rank-sum test, was used to identify differentially expressed genes between SARS-CoV-2-binding B cells and non-SARS-CoV-2-binding B cells within each transcriptionally-defined cluster. VDJ data were processed via Cell Ranger, IMGT/HighV-QUEST, and CHANGE-O, as outlined above to assign V-genes, and isotypes, and calculate percent VH somatic hypermutation.

### Reporting summary

Further information on research design is available in the Nature Research Reporting Summary linked to this article.

## Supplementary information

Supplementary Information

Description of Additional Supplementary Files

Supplementary Data 1

Reporting Summary

## Data Availability

The sequences for monoclonal antibodies characterized in this study have been deposited in GenBank under accession numbers OK157872, OK157873, OK157874, OK157875: The raw single-cell RNA sequencing data in this study have been deposited in the Sequence Read Archive under BioProject accession number PRJNA762922: The CyTOF FCS files generated in this study have been depositied in the Flow Repository under the following links: Fig. 2 Data: https://flowrepository.org/experiments/4853 ID: FR-FCM-Z4NL) Fig. 4 Data: https://flowrepository.org/experiments/4854 (ID: FR-FCM-Z4NM) Supplementary Figs. 1–4 Data: https://flowrepository.org/experiments/4857 (ID: FR-FCM-Z4NP). All other data are available in the article and its Supplementary files or from the corresponding author upon reasonable request. Source data are provided with this paper.

## Source data

Source Data
