# Supplementary material for: Single-cell profiling of the antigen-specific response to BNT162b2 SARS-CoV-2 RNA vaccine
Source: Nat Commun. 2022 Jun 16;13:3466. doi: 10.1038/s41467-022-31142-5 (PMC9201272; doi:10.1038/s41467-022-31142-5)
Supplement: Supplementary file 1 — Supplementary Information [file 41467_2022_31142_MOESM1_ESM.pdf]

SUPPLEMENTARY FIGURES

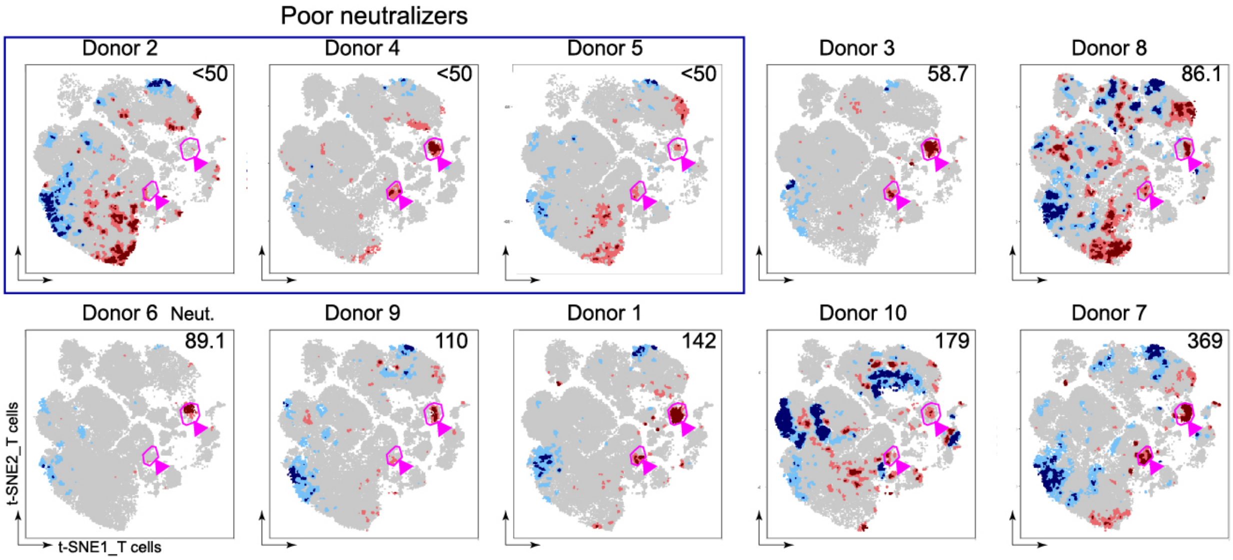

Supplementary Figure 1. T-REX analysis of T cell populations for individual donors. Individual t-SNE MAPS are shown for each donor. T-REX analysis showing contracting populations (blue) and expanding populations (red) for T cells pre- and post-BNT162b2 vaccination. Pink circles denote the antigen-specific $CD8^{+}CD38^{+}ICOS^{+}$ T cells (right) and $CD4^{+}CD38^{+}ICOS^{+}$ T cells (middle) identified in Figure 1. Plasma dilution necessary to achieve 50% neutralization in a SARS-CoV-2 vesicular stomatitis virus pseudonormalization assay is shown in the top right of each map. Donors who failed to achieve 50% neutralization with a 50-fold dilution were deemed poor neutralizers.

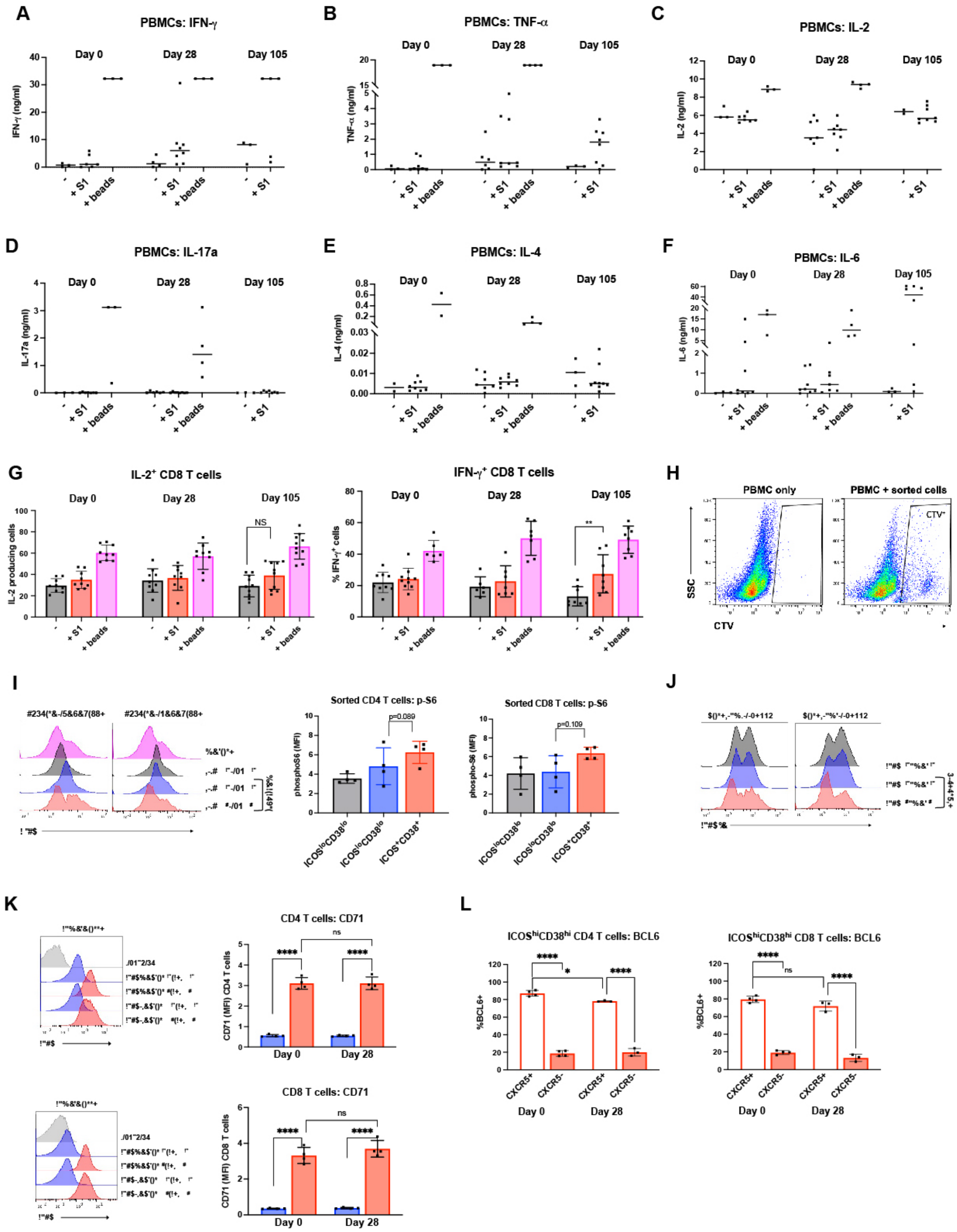
A
PBMCs: IFN-γ
Day 0
Day 28
Day 105
IFN-γ (ng/ml)
+ S1
+ beads
B
PBMCs: TNF-α
TNF-α (ng/ml)
C
PBMCs: IL-2
IL-2 (ng/ml)
D
PBMCs: IL-17a
IL-17a (ng/ml)
E
PBMCs: IL-4
IL-4 (ng/ml)
F
PBMCs: IL-6
IL-6 (ng/ml)
G
IL-2+ CD8 T cells
IL-2 producing cells
NS
IFN-γ+ CD8 T cells
% IFN-γ+ cells
**
H
PBMC only
PBMC + sorted cells
CTV+
SSC
CTV
I
Sorted CD4 T cells: p-S6
phosphoS6 (MFI)
p=0.089
Sorted CD8 T cells: p-S6
phospho-S6 (MFI)
p=0.109
ICOSloCD38lo
ICOSloCD38lo
ICOS+CD38+
J
K
CD4 T cells: CD71
CD71 (MFI) CD4 T cells
****
ns
CD8 T cells: CD71
CD71 (MFI) CD8 T cells
L
ICOShiCD38hi CD4 T cells: BCL6
%BCL6+
*
ICOShiCD38hi CD8 T cells: BCL6
CXCR5+
CXCR5-

Supplementary Figure 2. Cytokine and stimulation assays for expanding T cell populations. A-G. PBMCs collected from study participants pre-vaccination (day 0), day 28 post-vaccination, and day 100 post-vaccination were cultured with no stimulation (-), Spike protein (+S1), or polyclonal anti-CD2/3/28 beads. (**A-F**) Supernatants were collected from cultures after 4 days and predicted concentrations of each cytokine was determined by Legendplex bead capture assay. Each data point represents an individual participant. (**G**) PBMC Cultures were maintained until day 11 post-stimulation and then subjected to flow cytometry to determine percentages of IL-2 and IFN-γ producing T cells. Two-way ANOVA with Sidak's multiple comparisons test. Each data point represents an individual participant. **H.** Representative example of CellTraceViolet (CTV)-labeled samples from FACS, reintroduced to T cell-depleted autologous unlabeled PBMC cultures (n=4 independent donors). **I.** Sorted CTV^{+} samples were left unstimulated (grey) or stimulated with SARS-CoV-2 peptide (blue and red) for 2 days and phospho-S6 was measured by flow cytometry. Significance was determined by two-way paired ANOVA. Each data point represents an individual participant. **J.** Representative Glut-1 staining in sorted samples as in I (n=4). **K, L**. Day 0 and day 28 PBMCs were analyzed for (**K**) CD71 and (**L**) the percentage of sorted $ICOS^{+}CD38^{+}$ and $ICOS^{lo}CD38^{lo}$ $CD4^{+}$ and $CD8^{+}$ T cells positive for BCL6 according to CXCR5 expression. Two-way ANOVA with Sidak's multiple comparisons test and means ± SD are shown. Each data point represents an individual participant. ns= not significant, ** p<0.005, ****p<0.00005.

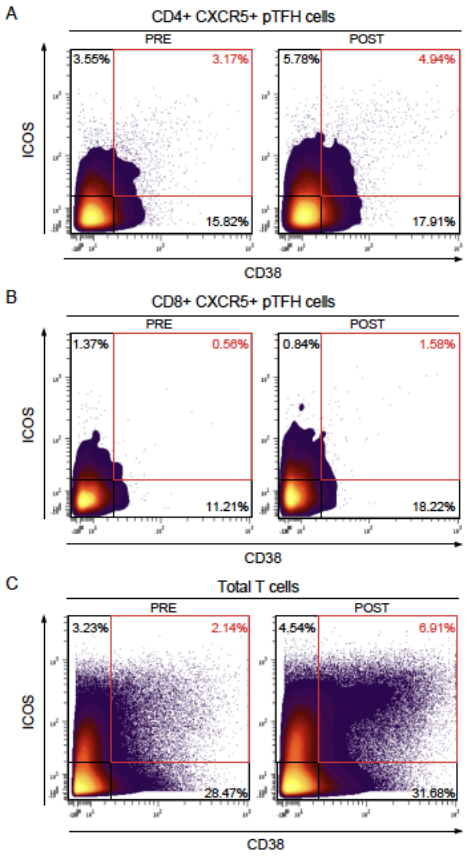

Supplementary Figure 3. Analysis of CyTOF data for pTfh cells. Representative data collected for Figure 2 was manually gated for $CD4^+$ or $CD8^+$ and $CXCR5^+$ to measure $ICOS^+CD38^+$ cells and total T cell $ICOS^+CD38^+$ frequency.

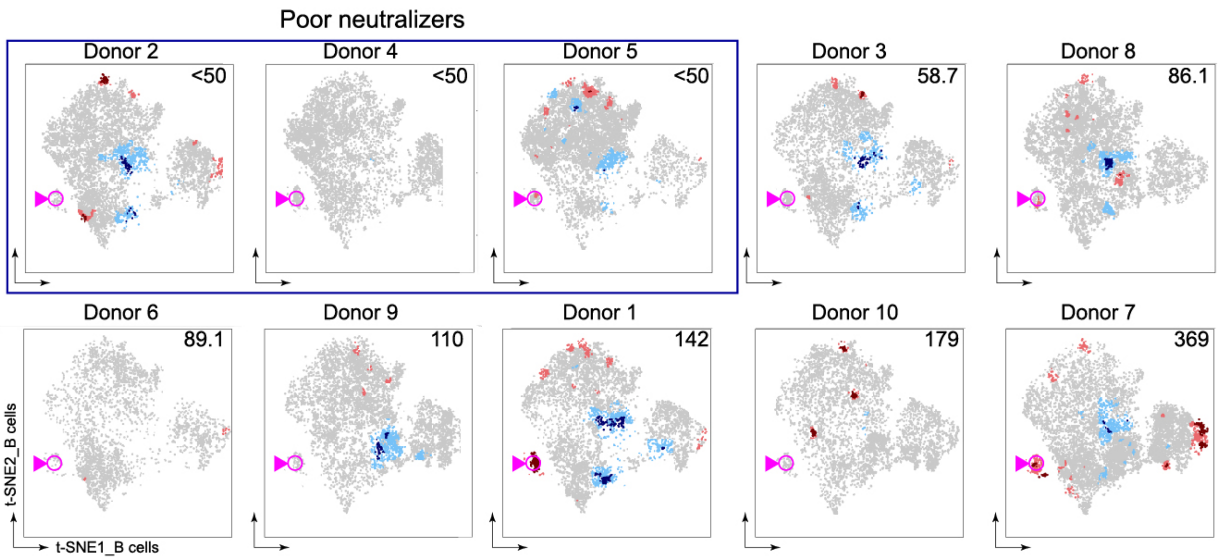

Supplementary Figure 4. T-REX analysis of B cell populations for individual donors. Individual t-SNE MAPS are shown for each donor. T-REX analysis showing contracting populations (blue) and expanding populations (red) for B cells pre- and post-BNT162b2 vaccination. Pink circles denote the activated plasmablast population. Plasma dilution necessary to achieve 50% neutralization in a SARS-CoV-2 vesicular stomatitis virus pseudonormalization assay is shown in the top right of each map. Donors who failed to achieve 50% neutralization with a 50-fold dilution were deemed poor neutralizers.

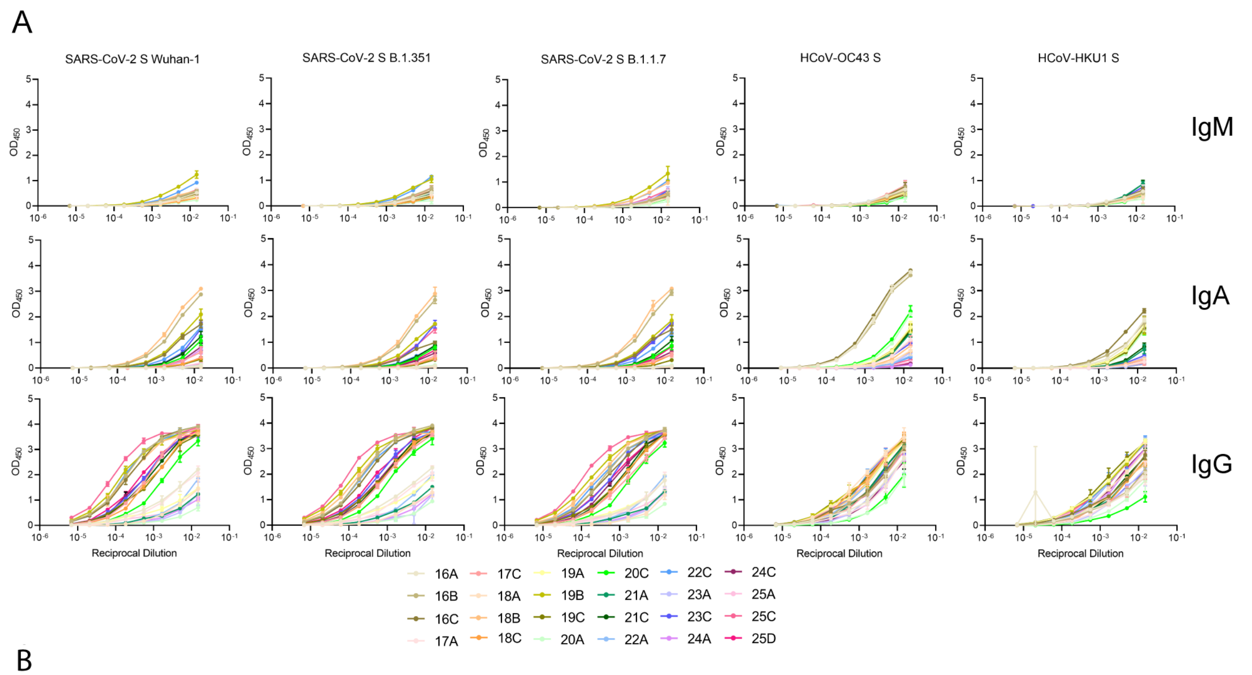

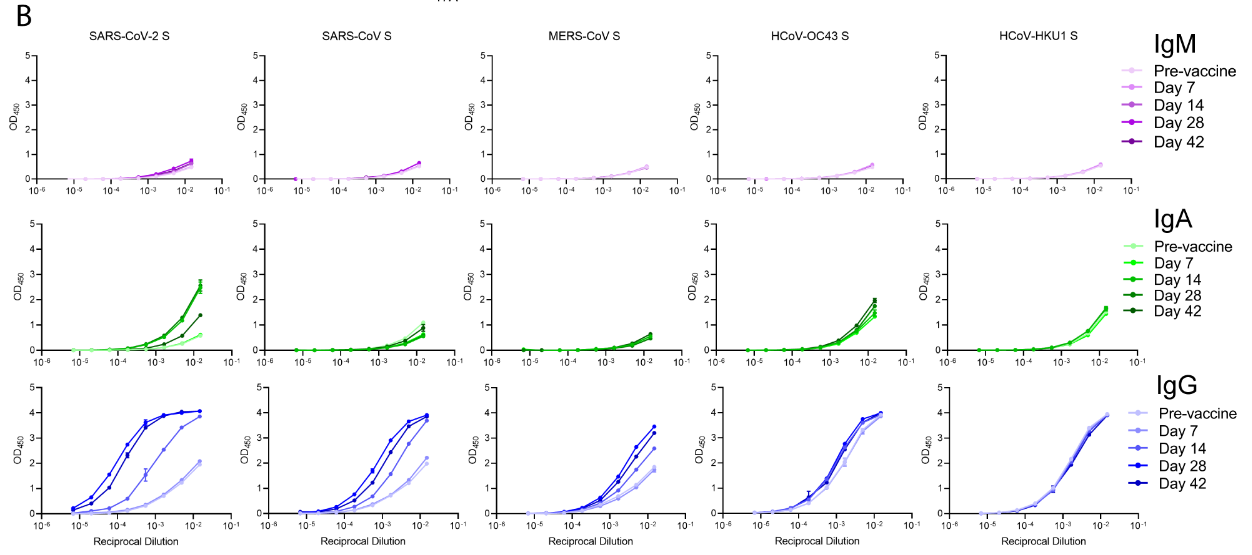

Supplementary Figure 5. ELISA data for individual donors. A. ELISA curves for 10 donor vaccinee plasma for IgM, IgA, and IgG isotypes against spike proteins from SARS-CoV-2, SARS-CoV-2 B.1.351, SARS-CoV-2 B.1.1.7, HCoV-OC43, and HCoV-HKU1. The optical density at 450 nm (y-axis) is plotted as a function of plasma dilution (x-axis). Each curve corresponds to a specific donor and timepoint, denoted with the donor number followed by the respective timepoint: A, pre-vaccine; B, 7-10 days post boost; C, D 105 days post boost. **B.** ELISA curves for longitudinal donor plasma for IgM, IgA, and IgG isotypes against spike

proteins from SARS-CoV-2, SARS-CoV, MERS-CoV, HCoV-OC43, and HCoV-HKU1. The optical density at 450 nm (y-axis) is plotted as a function of plasma dilution (x-axis). Means ± SD are shown.

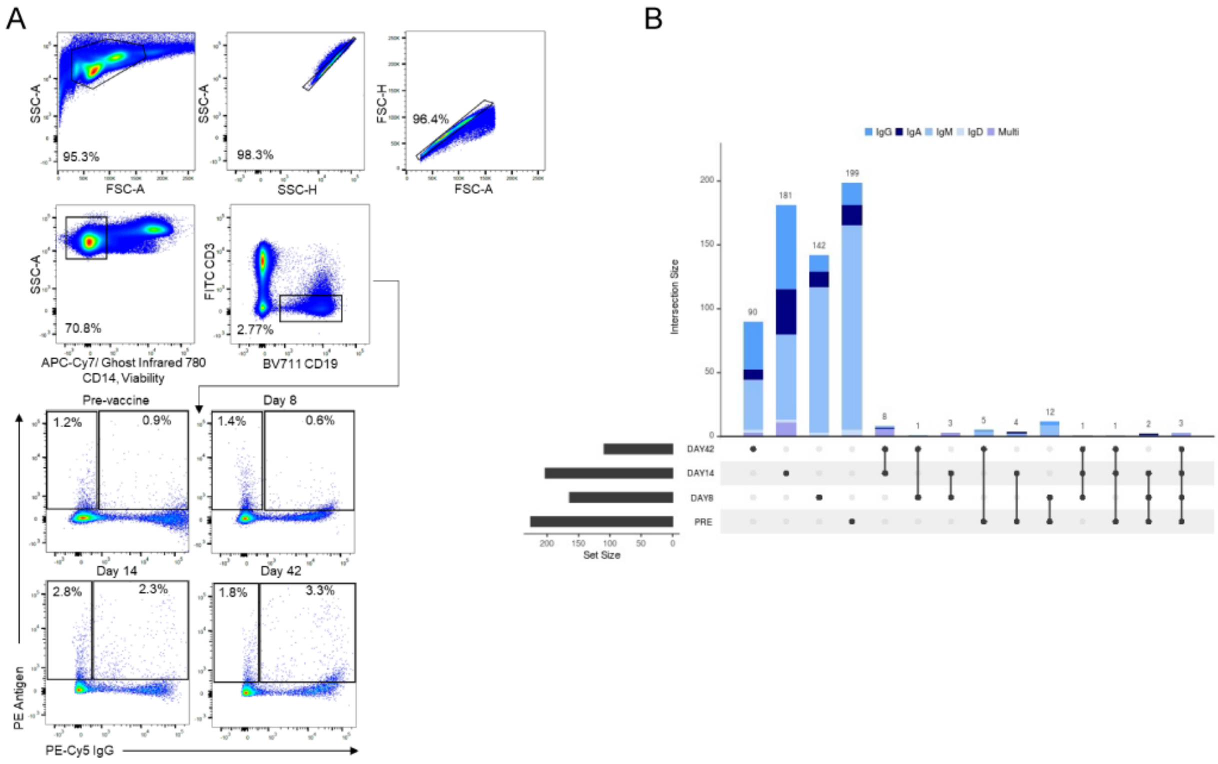

Supplementary Figure 6. Flow cytometry enrichment of coronavirus antigen-binding B cells. A. Gating scheme for fluorescence activated cell sorting (FACS) of PBMCs. Cells were stained with viability dye Ghost Infrared 780, CD14-APC-Cy7, CD3-FITC, BV711-CD19, and IgG-PE-Cy5. Antigen positive B cells were detected by streptavidin-PE binding to biotinylated DNA-oligo tagged antigens. Gates are drawn based on parameters set during cell sorting and percentages are from sort are listed in each plot. The gate for enrichment of CD19+ cells is shown for the pre-vaccine sample; Day 8, Day 14, and Day 42 samples all followed this sorting scheme. **B.** Representation of unique and shared clonotypes between different timepoints. For each individual and combination of timepoints, the number of clonotypes is displayed as a vertical bar graph. Shared clonotypes between timepoints is displayed by filled circles, showing which timepoints are part of a given shared cluster/combination where each combination is mutually exclusive. For each timepoint, the total number unique and shared clonotypes is indicated as a horizontal bar at the bottom left of the panel. Isotypes of clonotypes are

represented in different colors in the vertical bar graphs. Clusters with multiple isotypes is shown as "Multi."

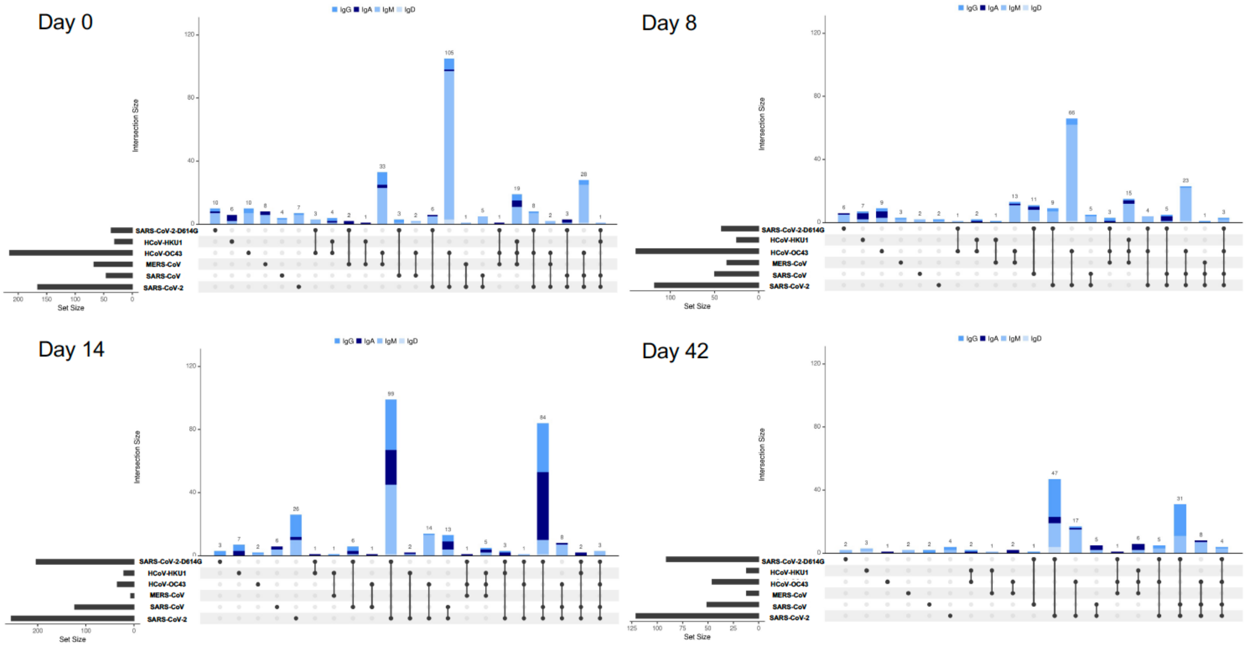

Supplementary Figure 7. B cell clones with cross-reactive LIBRA-seq scores over time. For each combination of coronavirus antigens, the number of B cells with high LIBRA-seq scores (≥1) is displayed as a bar graph. The combination of antigens is displayed by filled circles, showing which antigens are part of a given combination. Each combination is mutually exclusive. The number of B cells with high LIBRA-seq scores for each antigen is indicated as a horizontal bar at the bottom left of the panel. Isotypes of select B cells are depicted as different shades of blue beginning at IgD and following with IgM, IgG, and IgA.

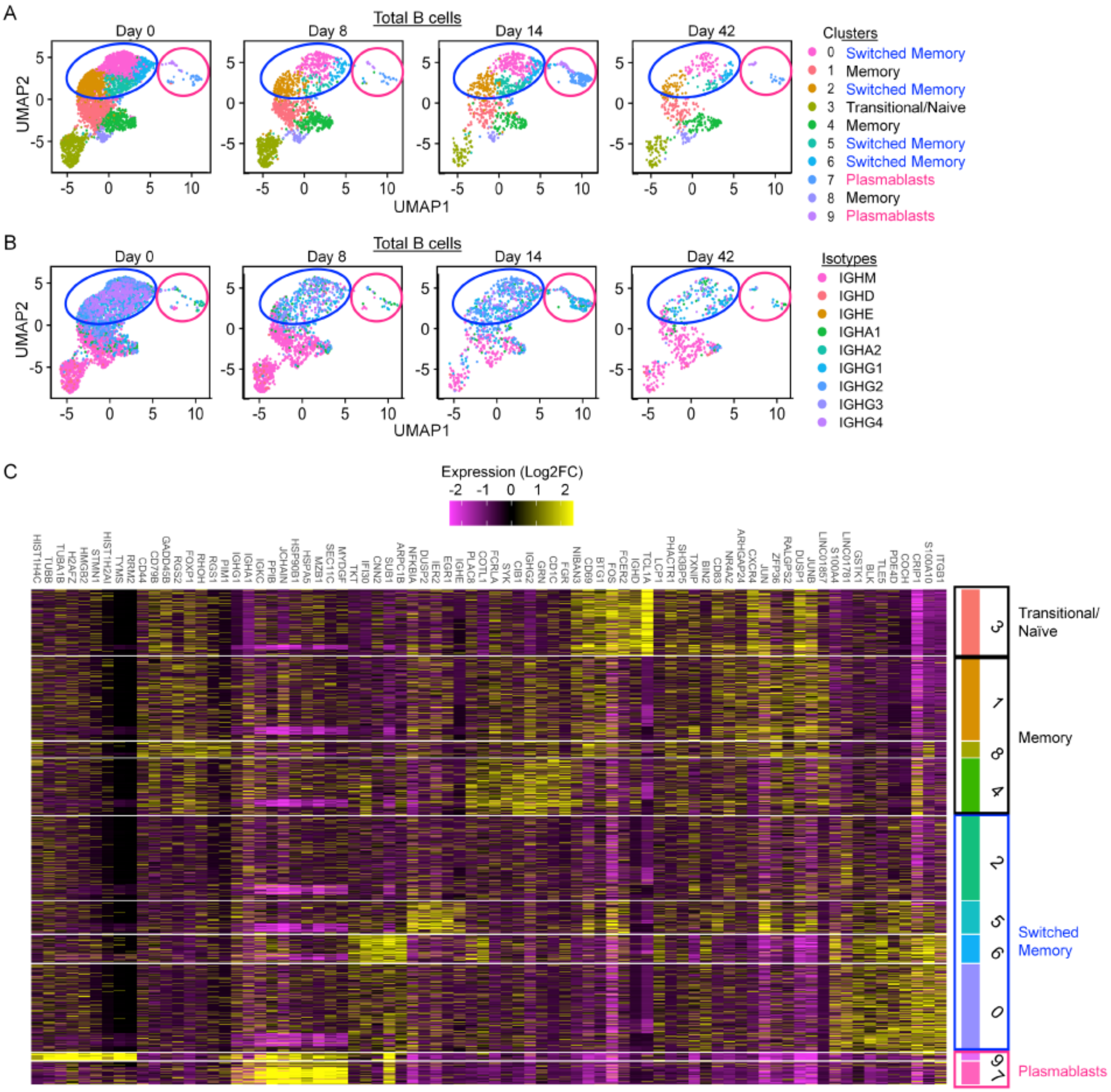

Supplementary Figure 8. Single-cell RNA-seq analysis identifies distinct clusters of memory B cell and plasmablast subsets. A, B. Single-cell RNA-seq analysis of B cells over time in which clusters were identified using Seurat and UMAP dimensionality reduction. (**A**) Clusters and (**B**) BCR isotypes are shown. **C**. The top 10 differentially expressed genes were identified by Seurat for each cluster. Heatmap depicts relative expression for each gene, columns are individual cells within each cluster.

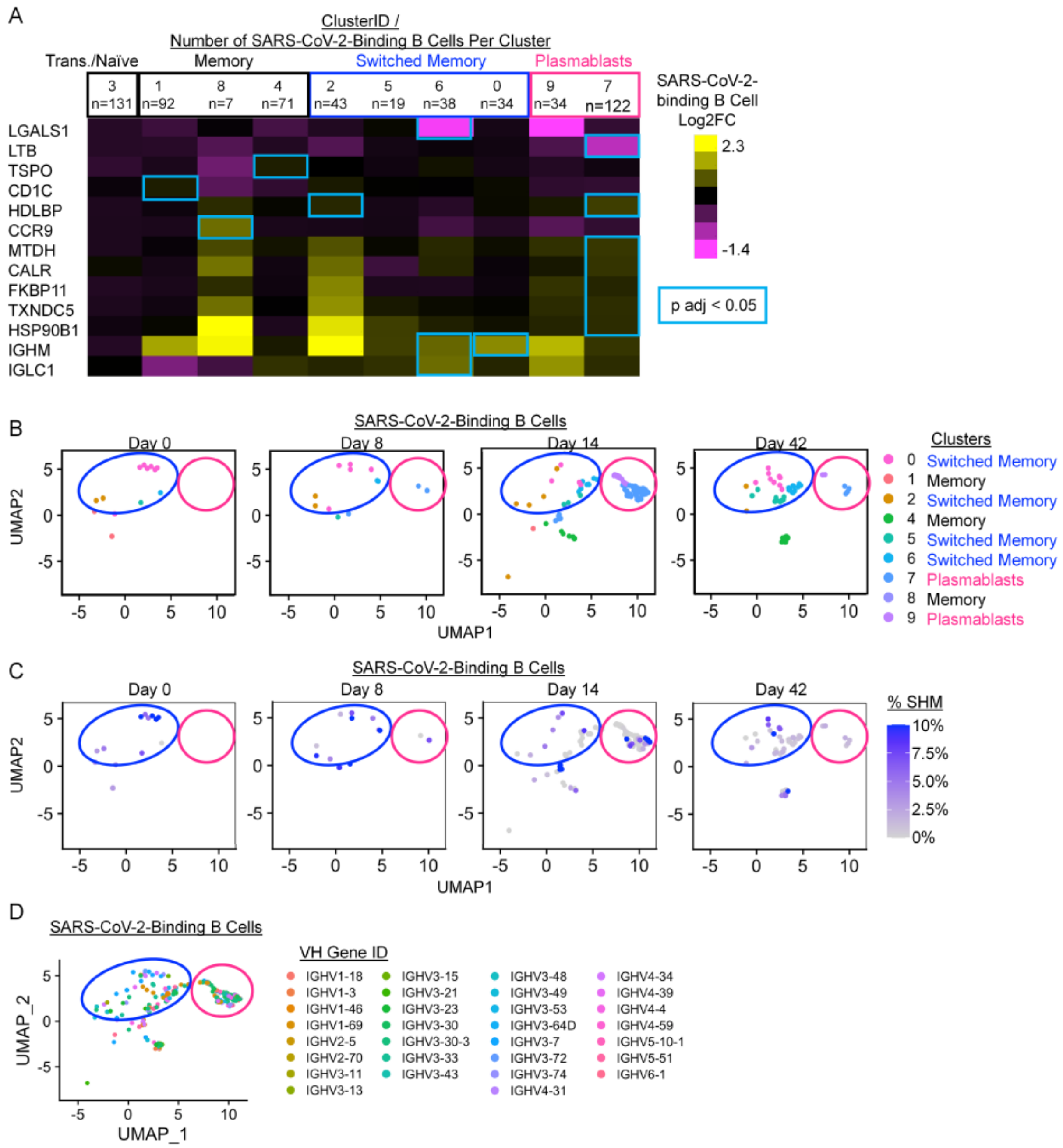

Supplementary Figure 9. Coronavirus antigen-binding B cell analyses. A. SARS-CoV-2-binding B cells were identified by LIBRAseq and were compared to non-SARS-CoV-2-binding B cells within each cluster. Log2 fold change is shown for differentially-expressed genes that were significantly different between these two groups in at least one cluster (p adj < 0.05, blue rectangles). **B-D**. SARS-CoV-2-binding B cells were analyzed over time for (**B**) cluster

assignments as defined in **Supplemental Figure 8**, (**C**) for percent somatic hypermutation (SHM), and (**D**) VH gene usage following vaccination with BNT162b2.

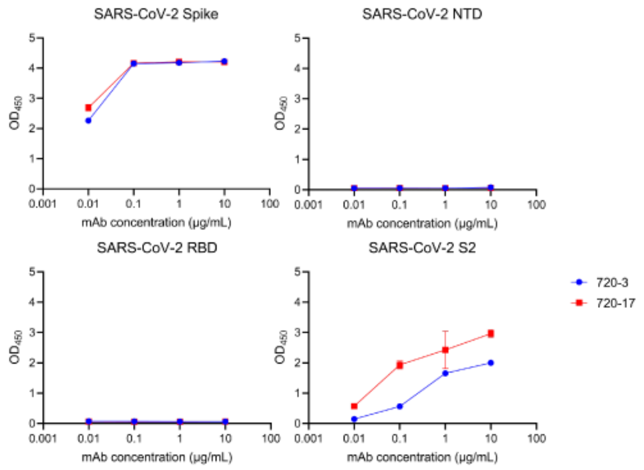

Supplementary Figure 10. Domain mapping of recombinant monoclonal antibodies.

ELISA data for the SARS-CoV-2 spike and the truncated SARS-CoV-2 NTD, RBD, and S2 domains. Optical density at 450 nm (y-axis) is depicted as a function of antibody concentration (x-axis). Means ± SD are shown.

Supplementary Table 1. Antibodies used in CyTOF analyses

T cell panel

Mass	Target	Clone	Vendor	Product #	Stain condition*, dilution	Custom conjugate
89	CD45	HI30	Fluidigm	3089003B	Ex, 1:200	
103	Live/dead	-	Fluidigm	201103B	Ex, 1:1000	
106	CD66b	80H3	Biolegend	305102	Ex, 1:100	X
110	CD16	3G8	Biolegend	302051	Ex, 1:100	X
111	CD8	RPAT8	Biolegend	301053	Ex, 1:100	X
112	CD14	M5E2	Biolegend	301843	Ex, 1:100	X
113	CD4	RPA-T4	Biolegend	300541	Ex, 1:100	X
114	CD3	UCHT1	Biolegend	300443	Ex, 1:100	X
116	CD19	HIB19	Biolegend	302247	Ex, 1:100	X
141	CD45R0	UCHL1	Biolegend	304239	Ex, 1:100	X
142	CPT1a	8F6AE9	Abcam	ab128568	Me, 1:100	X
143	CD127	A019D5	Fluidigm	3143012B	Ex, 1:200	
144	ATP5a	7H10BD4F9	Abcam	ab110273	Ex, 1:100	X
145	GRIM19	6E1BH7	Abcam	ab110240	Ex, 1:100	X
147	CD20	2H7	Fluidigm	3147001B	Ex, 1:200	
148	CD27	L128	BD	624084	Ex, 1:100	X
149	CCR4	205410	Fluidigm	3149029A	Ex, 1:200	
150	CD134	ACT35	Fluidigm	3150023C	Ex, 1:100	
151	ICOS	C398.4A	Fluidigm	3151020B	Ex, 1:200	
152	TCRgd	11F2	Fluidigm	3152008B	Ex, 1:200	
153	-	-	-	-	-	
154	GLUT3	polyclonal	Abcam	ab41525	Me, 1:100	X
155	-	-	-	-	-	
156	CXCR3	G025H7	Fluidigm	3156004B	Ex, 1:200	
158	CD137	4B4-1	Fluidigm	3158013B	Ex, 1:200	
159	CCR7	G043H7	Fluidigm	3159003A	Ex, 1:100	
160	CD98	MEM-108	Biolegend	315602	Ex, 1:100	X
161	CTLA4	14D3	Fluidigm	3161004B	Me, 1:100	
162	Ki-67	B56	Fluidigm	3162012B	Me, 1:200	
163	GLUT1	polyclonal	ovus Biologica	NB110-39113	Me, 1:100	X
164	CD95	DX2	Fluidigm	3164008B	Ex, 1:200	
166	CD44	BJ18	Fluidigm	3166001C	Ex, 1:100	
167	CD38	HIT2	Fluidigm	3167001B	Ex, 1:100	
168	CYTOC	6H2.B4	BD	556432	Me, 1:100	X
169	CD25	2A3	Fluidigm	3169003B	Ex, 1:200	
170	CD45RA	HI100	Fluidigm	3170010B	Ex, 1:200	
171	CXCR5	RF8B2	Fluidigm	3171014B	Ex, 1:200	
172	CD57	HCD57	Fluidigm	3172009B	Ex, 1:100	
173	CXCR4	12G5	Fluidigm	3173001B	Ex, 1:200	
174	HLA-DR	II L243	Fluidigm	3174001B	Ex, 1:200	
175	PD-1	EH12.2H7	Fluidigm	3175008B	Ex, 1:200	

176	CD56	CMSSB	Fluidigm	3176003B	Ex, 1:200	
191	DNA	-	Fluidigm	201192B	Me, 1:2000	
193	DNA	-	Fluidigm	201192B	Me, 1:2000	
209	CD11b	ICRF44	Fluidigm	3209003B	Ex, 1:200	

B cell panel

Mass	Target	Clone	Vendor	Product #	Stain condition*, dilution	Custom conjugate
89	CD45	HI30	Fluidigm	3089003B	Ex, 1:200	
103	Live/dead	-	Fluidigm	201103B	Ex, 1:1000	
106	CD66b	80H3	Biolegend	305102	Ex, 1:100	X
110	CD16	3G8	Biolegend	302051	Ex, 1:100	X
111	CD8	RPAT8	Biolegend	301053	Ex, 1:100	X
112	CD14	M5E2	Biolegend	301843	Ex, 1:100	X
113	CD4	RPA-T4	Biolegend	300541	Ex, 1:100	X
114	CD3	UCHT1	Biolegend	300443	Ex, 1:100	X
116	CD19	HIB19	Biolegend	302247	Ex, 1:100	X
141	CD45R0	UCHL1	Biolegend	304239	Ex, 1:100	X
142	CPT1a	8F6AE9	Abcam	ab128568	Me, 1:100	X
143	CD5	UCHT2	Fluidigm	3143007B	Ex, 1:100	
144	ATP5a	7H10BD4F9	Abcam	ab110273	Me, 1:100	X
145	GRIM19	6E1BH7	Abcam	ab110240	Me, 1:100	X
146	IgD	IA6-2	Fluidigm	3146005B	Ex, 1:200	
147	CD20	2H7	Fluidigm	3147001B	Ex, 1:200	X
148	CD27	L128	BD Custom	624084	Ex, 1:100	
150	CD43	84-3C1	Fluidigm	3150006B	Ex, 1:100	
151	IgL	MHL-38	Fluidigm	3151004B	Ex, 1:200	
152	CD21	BL13	Fluidigm	3152010B	Ex, 1:200	
153	CD62L	DREG-56	Fluidigm	3153004B	Ex, 1:200	
154	GLUT3	polyclonal	Abcam	ab41525	Me, 1:100	X
155	PD-1	EH12.2H7	Fluidigm	3155009B	Ex, 1:200	
156	CD86	IT2.2	Fluidigm	3156008B	Ex, 1:200	
158	-	-	-	-	-	
159	CD22	HIB22	Fluidigm	3159005B	Ex, 1:100	
160	CD98	MEM-108	Biolegend	315602	Ex, 1:100	X
161	IgK	MHK-49	Fluidigm	3160005B	Ex, 1:100	
162	CD79B	CB3-1	Fluidigm	3162008B	Ex, 1:400	
163	GLUT1	polyclonal	ovus Biologica	NB110-39113	Me, 1:100	X
165	CD40	5C3	Fluidigm	3165005B	Ex, 1:100	
166	CD44	BJ18	Fluidigm	3166001C	Ex, 1:100	
167	CD38	HIT2	Fluidigm	3167001B	Ex, 1:100	
168	CYTOC	6H2.B4	BD	556432	Me, 1:100	X
169	CD24	ML5	Fluidigm	3169004B	Ex, 1:200	
170	CD45RA	HI100	Fluidigm	3170010B	Ex, 1:200	
171	CXCR5	RF8B2	Fluidigm	3171014B	Ex, 1:200	

172	IgM	MHM-88	Fluidigm	3172004B	Ex, 1:100	
173	CXCR4	12G5	Fluidigm	3173001B	Ex, 1:200	
174	HLA-DR/MHC	II L243	Fluidigm	3174001B	Ex, 1:200	
175	CD71	OKT-9	Fluidigm	3175011B	Ex, 1:200	
176	CD56	CMSSB	Fluidigm	3176003B	Ex, 1:200	
191	DNA	-	Fluidigm	201192B	Me, 1:2000	
193	DNA	-	Fluidigm	201192B	Me, 1:2000	
209	CD11b	ICRF44	Fluidigm	3209003B	Ex, 1:200	

*Key to staining conditions:

Ex=extracellular, live cell stain

Me=stain post permeabilization with methanol
