# Supplementary material for: Single-cell profiling of the antigen-specific response to BNT162b2 SARS-CoV-2 RNA vaccine
Source: Nat Commun. 2022 Jun 16;13:3466. doi: 10.1038/s41467-022-31142-5 (PMC9201272; doi:10.1038/s41467-022-31142-5)
Supplement: Supplementary file 2 — Description of Additional Supplementary Files [file 41467_2022_31142_MOESM2_ESM.pdf]

Supplementary Data 1

B and T cell CyTOF data from from individual study participants in Figures 2 and 4 and Supplementary Figures 1 and 4 as analyzed for population abundance by expert gating or TREX. Pre-vaccine (day 0) and post-vaccine (day 28) population frequencies are shown and colored by relative abundance. Log2-fold change between pre- and post-vaccine population frequencies are shown at bottom and color coded for increased (red) or decreased (blue) abundance.
