# Supplementary material for: Single-cell profiling of the antigen-specific response to BNT162b2 SARS-CoV-2 RNA vaccine
Source: Nat Commun. 2022 Jun 16;13:3466. doi: 10.1038/s41467-022-31142-5 (PMC9201272; doi:10.1038/s41467-022-31142-5)
Supplement: Supplementary file 4 — Reporting Summary [file 41467_2022_31142_MOESM4_ESM.pdf]

nature portfolio

Corresponding author(s): Jeffrey C. Rathmell, Rachel H. Bonami, Ivelin S. Georgiev, Jonathan M. Irish

Last updated by author(s): Apr 5, 2022

Reporting Summary

Nature Portfolio wishes to improve the reproducibility of the work that we publish. This form provides structure for consistency and transparency in reporting. For further information on Nature Portfolio policies, see our Editorial Policies and the Editorial Policy Checklist.

Statistics

For all statistical analyses, confirm that the following items are present in the figure legend, table legend, main text, or Methods section.

n/a	Confirmed	
☐	☒	The exact sample size (n) for each experimental group/condition, given as a discrete number and unit of measurement
☐	☒	A statement on whether measurements were taken from distinct samples or whether the same sample was measured repeatedly
☐	☒	The statistical test(s) used AND whether they are one- or two-sided<br>*Only common tests should be described solely by name; describe more complex techniques in the Methods section.*
☐	☒	A description of all covariates tested
☐	☒	A description of any assumptions or corrections, such as tests of normality and adjustment for multiple comparisons
☐	☒	A full description of the statistical parameters including central tendency (e.g. means) or other basic estimates (e.g. regression coefficient) AND variation (e.g. standard deviation) or associated estimates of uncertainty (e.g. confidence intervals)
☐	☒	For null hypothesis testing, the test statistic (e.g. F, t, r) with confidence intervals, effect sizes, degrees of freedom and P value noted<br>*Give P values as exact values whenever suitable.*
☐	☒	For Bayesian analysis, information on the choice of priors and Markov chain Monte Carlo settings
☐	☒	For hierarchical and complex designs, identification of the appropriate level for tests and full reporting of outcomes
☐	☒	Estimates of effect sizes (e.g. Cohen's d, Pearson's r), indicating how they were calculated

Our web collection on statistics for biologists contains articles on many of the points above.

Software and code

Policy information about availability of computer code

Data collection	No specific software was used for data collection beyond equipment operating software provided by manufacturers according to each piece of equipment as detailed in methods.
Data analysis	All software are available. T-REX is available https://github.com/cytolab/T-REX; Marker Enrichment Modeling (MEM) https://github.com/cytolab/mem; Statistics were performed in GraphPad Prism 9.0; Flow cytometry was analyzed with FlowJo v10.6.2; Single cell B cell repertoire analyses were analyzed using Cell Ranger v5.0.0, HighV-Quest (Methods Mol Biol 882, 569-604 (2012)), Change-O (Bioinformatics 31, 3356-3358 (2015)), and LIBRA-seq (Cell 179, 1636-1646.e1615 (2019)); Single cell RNAseq analyses were performed using Seurat v4.0.0 (Nat Biotechnol 33, 495-502 (2015)). Antibody phylogenetic trees were visualized using Dendroscope (Syst Biol 61, 1061-1067 (2012)). Neutralization assays were analyzed with RTCA software version 2.1.0 (ACEA Biosciences Inc)

For manuscripts utilizing custom algorithms or software that are central to the research but not yet described in published literature, software must be made available to editors and reviewers. We strongly encourage code deposition in a community repository (e.g. GitHub). See the Nature Portfolio guidelines for submitting code & software for further information.

Data

Policy information about availability of data

All manuscripts must include a data availability statement. This statement should provide the following information, where applicable:

- Accession codes, unique identifiers, or web links for publicly available datasets
- A description of any restrictions on data availability
- For clinical datasets or third party data, please ensure that the statement adheres to our policy

Monoclonal antibodies sequences are available: BankIt2501068 Seq1, OK157872; BankIt2501068 Seq2, OK157873; BankIt2501068 Seq3, OK157874; BankIt2501068

Seq4, OK157875.
Single Cell RNAseq data are available as BioProject PRJNA762922 with link: https://www.ncbi.nlm.nih.gov/bioproject/?term=PRJNA762922
Mass cytometry datasets in this manuscript have been deposited in FlowRepository (http://flowrepository.org/). The FCS files from CyTOF analyses have been uploaded to Flow Repository under the following links:
Figure 2 Data: https://flowrepository.org/experiments/4853 ID: FR-FCM-Z4NL)
Figure 4 Data: https://flowrepository.org/experiments/4854 (ID: FR-FCM-Z4NM)
Supplemental Figures 1, 3, and 4 Data: https://flowrepository.org/experiments/4857 (ID: FR-FCM-Z4NP)

Field-specific reporting

Please select the one below that is the best fit for your research. If you are not sure, read the appropriate sections before making your selection.

- [x] Life sciences
- [ ] Behavioural & social sciences
- [ ] Ecological, evolutionary & environmental sciences

For a reference copy of the document with all sections, see nature.com/documents/nr-reporting-summary-flat.pdf

Life sciences study design

All studies must disclose on these points even when the disclosure is negative.

Sample size	The sample size was determine based on availability of donors and balancing depth and expense of analyses vs sample number
Data exclusions	None
Replication	Research assays performed on human samples were conducted as a single batch according to best practices in the field using a pair of samples from N=10 independent individuals run on multiple assays that included both redundant and complementary tests (i.e., CyTOF T cell panel, CyTOF B cell panel, and fluorescence flow cytometry). While sample availability limited most assays to be run as single batch, antibodies overlap between panels allowed for some antigens and cell types to be measured two or three times. Validations included synthesizing antibodies based on sequence data to confirm specificity to SARS-CoV-2 and cell sorting from samples based on predicted cell surface markers to test and confirm T cell specificity to Spike protein.
Randomization	These were longitudinal samples
Blinding	All donors received vaccine for this study. Donors were de-identified.

Reporting for specific materials, systems and methods

We require information from authors about some types of materials, experimental systems and methods used in many studies. Here, indicate whether each material, system or method listed is relevant to your study. If you are not sure if a list item applies to your research, read the appropriate section before selecting a response.

Materials & experimental systems

n/a	Involved in the study	
[ ]	[x]	Antibodies
[ ]	[x]	Eukaryotic cell lines
[x]	[ ]	Palaeontology and archaeology
[x]	[ ]	Animals and other organisms
[ ]	[x]	Human research participants
[x]	[ ]	Clinical data
[x]	[ ]	Dual use research of concern

Methods

n/a	Involved in the study	
[x]	[ ]	ChIP-seq
[ ]	[x]	Flow cytometry
[x]	[ ]	MRI-based neuroimaging

Antibodies

Antibodies used	CD8a e450 (Invitrogen 48-0086-42, 1:200) ICOS BV605 (Biolegend 313538, 1:50), CCR7 PE (Biolegend 353204, 1:200), CD38 PerCP (Biolegend 303520, 1:100), CD4 PECy7 (Biolegend 357410, 1:100), and CD3 APCCy7 (Biolegend 300318, 1:200), TNF-a AF488 (Biolegend 502915, 1:100), IFN-g APC (Invitrogen 17-7319-82, 1:100) IL-2 AlexaFluor 700 (Biolegend 500320, 1:150), granzyme b FITC (Biolegend 515403, 1:100), phospho-S6 APC Ser235/236 (Invitrogen 17-9007-42, 1:80), Bcl-6 FITC (Biolegend 358513, 1:100), CD71 (BioLegend 334108 1:100), and Glut-1 AlexaFluor 647 (Abcam ab115730, 1:300). Viability dye (Ghost Red 780, Tonbo Biosciences, 13-0865, 1 µL per 10e6 total cells), CD14-APC-Cy7 (BD, 561709, 1 µL per 60e6 total cells), CD3-FITC (Tonbo Bioscience, 35-0037, 1 µL per 20e6 total cells), CD19-BV711 (BD, 563036, 1 µL per 10e6 total cells), and IgG-PE-Cy5 (BD, 551497, 3 µL per 10e6 total cells). Streptavidin-PE (Invitrogen, S886, 1 µL per 100e6 total cells). Antibodies for CyTOF are listed in Supplemental Table 1
Validation	All antibodies for cell staining are commercially available. Antibody sequences are available for custom antibodies (see data reporting)

Commercial antibodies were tested at the manufacturer recommended amount as well as additional titrations to capture separation of negative and positive cellular populations. Custom conjugations of commercial antibodies were titrated against known positive and negative samples.

Eukaryotic cell lines

Policy information about cell lines

Cell line source(s)	FreeStyle293F cells (Thermo Fisher)
Authentication	purchased from vendor
Mycoplasma contamination	vendor
Commonly misidentified lines (See ICLAC register)	*Name any commonly misidentified cell lines used in the study and provide a rationale for their use.*

Human research participants

Policy information about studies involving human research participants

Population characteristics

The effect of BNT162b2 immunization was first explored on recipient T cell populations in a cohort of ten healthy donors who had not been previously infected with SARS-CoV-2. Donors had an average age of 41.8 ± 6.3 years (range 35-57). Six donors were male, and nine donors identified as having Caucasian ancestry. An additional single healthy male Caucasian donor of age 45-50 with no prior history of SARS-CoV-2 infection was recruited in a second longitudinal study.

Recruitment

Volunteer donors at Vanderbilt University Medical Center late in 2020 and early in 2021. Participants were recruited via email listserv in December 2020 in the United States. Informed consent was obtained, and a baseline health questionnaire including knowledge of prior SARS-CoV-2 infection was also completed. Participants were offered an Amazon gift card as compensation for their time. All participants were healthy and no source of selection bias was present as all donors received a normal vaccine course.
Simple phlebotomy was performed either pre-vaccine (day 0), day 28-30, and day 95-100 OR pre-vaccine and days 8, 14, and 42 after initial BNT162b2 vaccination using sodium citrate mononuclear cell preparation (CPT) tubes. All participants received two doses of vaccine 21 days apart.

Ethics oversight

IRB approval was obtained (VUMC 191562)

Note that full information on the approval of the study protocol must also be provided in the manuscript.

Flow Cytometry

Plots

Confirm that:

- [x] The axis labels state the marker and fluorochrome used (e.g. CD4-FITC).
- [x] The axis scales are clearly visible. Include numbers along axes only for bottom left plot of group (a 'group' is an analysis of identical markers).
- [x] All plots are contour plots with outliers or pseudocolor plots.
- [x] A numerical value for number of cells or percentage (with statistics) is provided.

Methodology

Sample preparation

Simple phlebotomy was performed using sodium citrate mononuclear cell preparation (CPT) tubes. CPT tubes were spun at 1600 RCF for 20 minutes. The plasma layer was carefully removed, transferred to a conical vial, spun at 600g for 10 minutes, and the supernatant transferred to microtubes in 1 mL aliquots. Plasma was stored at -80°C until further use. Buffy coat was divided amongst two clean conical tubes. CPT tubes were rinsed with 1 mL PBS (ThermoFisher, 10010-049), and the total volume in the conical was increased to 15cc. Cells were pelleted at 600g for 10 minutes. Cell pellets were combined and washed with 10cc PBS, and then cells were pelleted again at 600g for 10 minutes. PBS was discarded, and the pellet was re-suspended in 3mL ACK buffer (ThermoFisher, A1049201) for 5 minutes. 10 mL of PBS was added to the ACK cell suspension, and cells were pelleted for 10 minutes at 600g. Cells were resuspended in PBS, strained through a cell strainer (Falcon, 352235) and counted using an ACT Diff hematology analyzer (Beckman Coulter). Cells were pelleted by centrifugation at 600g for 10 minutes and resuspended in heat-inactivated FBS (GemCell, 100-500) containing 10% DMSO (Sigma-Aldrich, D2650) at a concentration of 5 million cells/mL in cryovials (ThermoFisher, 5000-0020). Cryovials were frozen overnight to -80°C using Mr. Frosty freezing containers (ThermoFisher, 5100-0050) and then transferred to liquid nitrogen for long-term storage.

Mass Cytometry:
Cells were incubated with a viability reagent (Cell ID Intercalator-Rh; Fluidigm), per the product literature. Then cells were washed in PBS without calcium or magnesium (Gibco, Thermo Fisher Scientific) containing 1% BSA (Thermo Fisher Scientific) and stained in 50 μL PBS and BSA 1%–containing antibody cocktail for extracellular targets. Cells were stained for 30 minutes at room temperature using the antibodies listed in Supplemental Table 3. Cells were washed in PBS and BSA 1% and then

fixed with 1.6% paraformaldehyde (Electron Microscopy Sciences). Cells were washed once in PBS and permeabilized by resuspension in ice-cold methanol. After incubation overnight at −20°C, cells were washed with PBS and BSA 1% and stained in 50 μL PBS and BSA 1%–containing antibody cocktail for intracellular targets. Cells were washed in PBS and BSA 1%, then washed with PBS and stained with an iridium DNA intercalator (Fluidigm) for 20 minutes at room temperature. Finally, cells were washed with PBS and with diH2O before being resuspended in 1× EQ Four Element Calibration Beads (Fluidigm) and collected on a Helios mass cytometer (Fluidigm) at the Vanderbilt Flow Cytometry Shared Resource Center.

Fluorescence flow cytometry:
For cell surface stains PBMCs were first resuspended with Human TruStain Fcx (Biolegend) for 10 minutes at room temperature and then stained with indicated antibodies for 20-30 minutes in PBS+2%FCS, washed and resuspended in PBS +2%FCS for analysis. For Intracellular cytokine staining cells were either stimulated with PMA (1ug/ml) and ionomycin (750ng/ml) for 5 hours, or restimulated on day 11 in 96-well plates with 2.5ug/ml recombinant SARS-CoV-S protein S1 (Biolegend 792906, carrier-free) for 8h in the presence of 1ug/ml Golgiplug and 0.7ug/ml Golgistop. Peptivator-stimulated cultures were treated with Golgiplug/Golgistop overnight after 2 days of activation. Cells were surface stained with indicated antibodies in FACS buffer, fixed with 1.5% paraformaldehyde for 10 minutes, and permeabilized with methanol for 20 minutes on ice.

Cell Sorting for LIBRAseq:
Cells were stained and mixed with DNA-barcoded antigens and other antibodies, and then sorted using fluorescence activated cell sorting (FACS). First, cells were counted and viability was assessed using Trypan Blue. Then, cells were washed three times with DPBS supplemented with 0.1% Bovine serum albumin (BSA). Cells were resuspended in DPBS-BSA and stained with cell markers including viability dye (Ghost Red 780), CD14-APC-Cy7, CD3-FITC, CD19-BV711, and IgG-PE-Cy5. Additionally, antigen-oligo conjugates were added to the stain (1 μg of every antigen except for HA NC99 and HCoV-HKU1 S which were added at 0.1 μg). After staining in the dark for 30 minutes at room temperature, cells were washed three times with DPBS-BSA at 300 g for five minutes. Cells were then incubated for 15 minutes at room temperature with Streptavidin-PE to label cells with bound antigen. Cells were washed three times with DPBS-BSA, resuspended in DPBS, and sorted by FACS

Instrument

Helios mass cytometer (Fluidigm), Miltenyi MACSQuant16 Analyzer, BD FACSAria III

Software

FlowJo v10.6.2, T-REX is available https://github.com/cytolab/T-REX; Marker Enrichment Modeling (MEM) https://github.com/cytolab/mem;

Cell population abundance

Population abundances are shown in gated populations as indicated in each relevant figure.

Gating strategy

Flow cytometry data were gating on singlets and bimodal populations and divided into bimodal populations where possible. Fluorescence minus one (FMO) or isotype controls were used to establish negative populations for other gating and for sorting for Figure 3 in Supplemental Figure 2.
Gating strategy for LIBRA-seq and single cell RNAseq for isolation of antigen-specific B cells was performed by collecting an initial 10,000 events on the cell sorter and partitioning based on the signal separation of the negative and positive populations. Because we used an NGS-based readout for antigen reactivity (LIBRA-seq), the antigen-specific detection gate (PE) was set loosely in order to acquire potentially low affinity pre-cursor B cells which could be present close to the non-antigen specific B cell population. Further, false positive antigen-specific B cells sorted by fluorescence signal are discarded through NGS data filtering because of no associated antigen barcode upon single cell encapsulation. Shown in Supplemental Figure 6

☒ Tick this box to confirm that a figure exemplifying the gating strategy is provided in the Supplementary Information.
